# New remarkably complete skeleton of *Mixodectes* reveals arboreality in a large Paleocene primatomorphan mammal following the Cretaceous-Paleogene mass extinction

**DOI:** 10.1038/s41598-025-90203-z

**Published:** 2025-03-11

**Authors:** Stephen G. B. Chester, Thomas E. Williamson, Jordan W. Crowell, Mary T. Silcox, Jonathan I. Bloch, Eric J. Sargis

**Affiliations:** 1 Department of Anthropology, Brooklyn College, City University of New York, 2900 Bedford Avenue, 11210 Brooklyn, NY USA; 2 PhD Program in Anthropology, The Graduate Center, City University of New York, 365 Fifth Avenue, 10016 New York, NY USA; 3 New York Consortium in Evolutionary Primatology, 10024 New York, NY USA; 4 New Mexico Museum of Natural History and Science, 1801 Mountain Road, NW, 87104-1375 Albuquerque, NM USA; 5 Department of Anthropology, University of Toronto Scarborough, 1265 Military Trail, M1C 1A4 Scarborough, ON Canada; 6 Florida Museum of Natural History, University of Florida, 1659 Museum Road, 32611-7800 Gainesville, FL USA; 7 Department of Anthropology, Yale University, P. O. Box 208277, 06520 New Haven, CT USA; 8 Divisions of Vertebrate Paleontology and Vertebrate Zoology, Yale Peabody Museum, 06520 New Haven, CT USA; 9 Yale Institute for Biospheric Studies, 06520 New Haven, CT USA

**Keywords:** Mixodectidae, Plesiadapiforms, Evolution, Postcranium, Locomotion, Palaeontology, Phylogenetics, Palaeoecology

## Abstract

Mixodectids are poorly understood placental mammals from the Paleocene of western North America that have variably been considered close relatives of euarchontan mammals (primates, dermopterans, and scandentians) with hypothesized relationships to colugos, extinct plagiomenids, and/or microsyopid plesiadapiforms. Here we describe the most complete dentally associated skeleton yet recovered for a mixodectid, specifically *Mixodectes pungens* from the early Paleocene of the San Juan Basin, New Mexico. A partial skull with all the teeth erupted and associated axial skeleton, forelimbs, and hind limbs, with epiphyses fused, indicate that it was a mature adult. Results from cladistic analyses incorporating new data robustly support primatomorphan (Primates + Dermoptera) affinities of Mixodectidae, but relationships within Euarchonta are less clear, with *Mixodectes* recovered as a stem primatomorphan, stem dermopteran, or stem primate. Analyses of postcrania suggest that *M. pungens* was a relatively large (~ 1.3 kg), claw-climbing arborealist capable of frequent clinging on large diameter vertical supports. With teeth suggesting an omnivorous diet that included leaves, *M. pungens* occupied a unique ecological niche in the early Paleocene of North America that differed from contemporary, arboreal plesiadapiforms that were smaller and more frugivorous. Euarchontans were thus a more diverse radiation in the early Cenozoic than previously appreciated.

**Supplementary Information:**

The online version contains supplementary material available at 10.1038/s41598-025-90203-z.

## Introduction

Mixodectids are a poorly defined group of enigmatic placental mammals from the Paleocene of western North America whose evolutionary relationships have long been hard to resolve due to their sparse fossil record that consists mostly of dental and gnathic remains^1,2^. Taxa classified as mixodectids have been linked to extant mammalian groups including Primates^1^, Rodentia^3^, “Insectivora”^4,5^, and Dermoptera^6^. The family Mixodectidae includes two species of *Mixodectes* from the Torrejonian North American Land Mammal Age (NALMA) of New Mexico (and possibly Wyoming); the monotypic *Dracontolestes* from the Torrejonian NALMA of Utah; and three species of *Eudaemonema* from the Torrejonian and Tiffanian NALMAs of Wyoming, Montana, and Alberta^7,8^. Similarities between the dentition of mixodectids and those of other extinct Paleogene families such as the Plagiomenidae and Microsyopidae have been recognized and debated for over a century^2–5,9,10^. These extinct families have also often been hypothesized to have affinities with one or more clades of euarchontan mammals (primates, colugos, treeshrews), but fossil evidence to test these hypotheses has been mostly limited to craniodental characters [e.g., ^11^]. Although fragmentary postcrania previously attributed to early Paleocene *Mixodectes* show similarities to those of other arboreal euarchontans^6,12,13^, more complete mixodectid specimens are needed to assess their significance regarding the early evolution of this group.

**Fig. 1:**
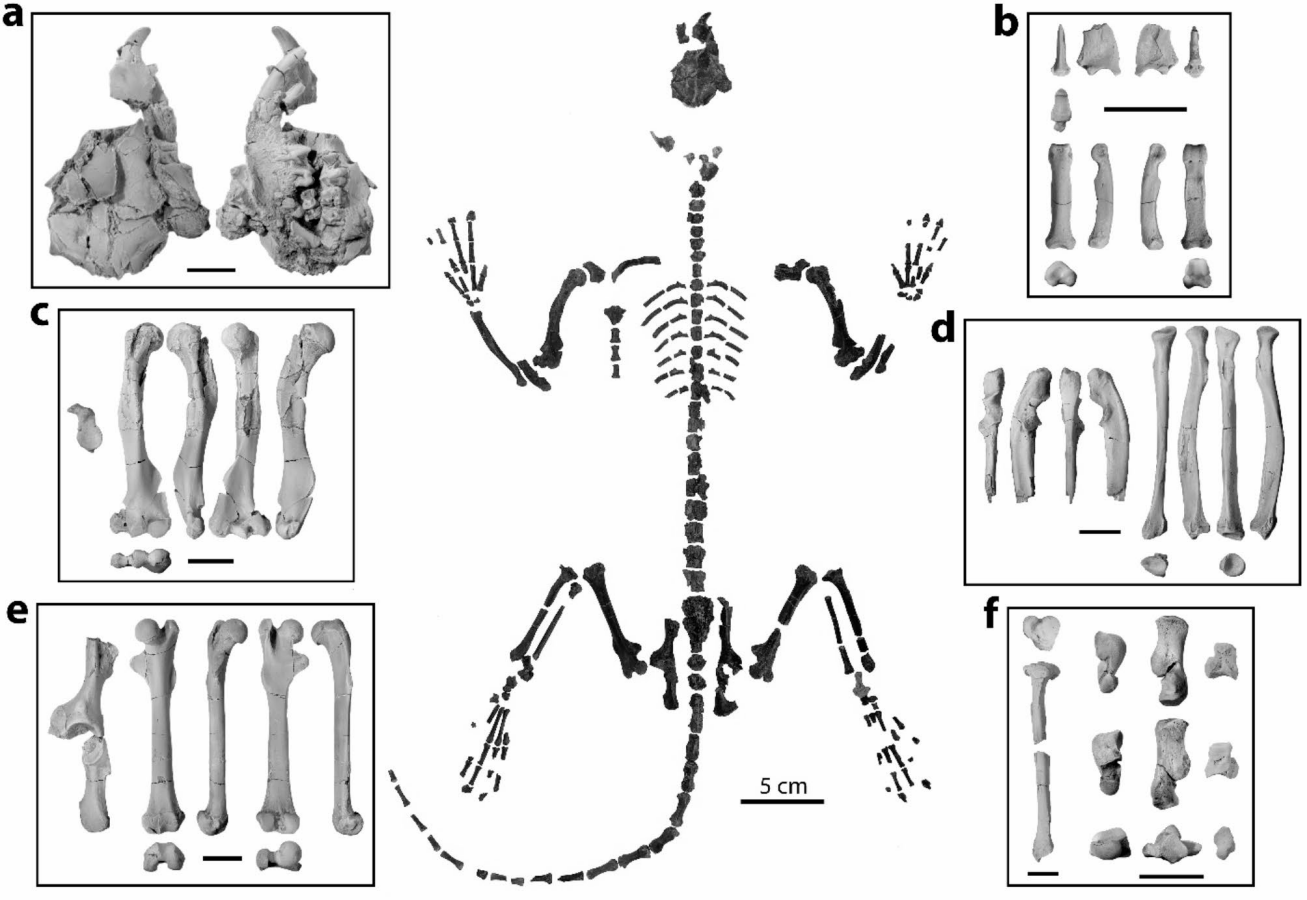
Skeleton composite of *Mixodectes pungens* (NMMNH P-54501) with most elements in ventral view or oriented to illustrate features such as articular surfaces. Many elements of the axial skeleton, manus, and pes could be identified to anatomical region but not to specific position and are presented to illustrate overall completeness of this specimen. Descriptions and orientations of skeletal elements in boxes A-F organized from left to right and then from top to bottom: (**a**) partial skull in dorsal, ventral views. (**b**) distal phalanx in dorsal, L lateral, R lateral, ventral, proximal views; proximal phalanx in dorsal, L lateral, R lateral, ventral, proximal, distal views. (**c**) L partial scapula in lateral view; L humerus in ventral, medial, dorsal, lateral, distal views. (**d**) R proximal ulna in ventral, lateral, dorsal, medial views; L radius in dorsolateral, dorsomedial, ventromedial, ventrolateral, distal, proximal views. (**e**) L partial innominate in lateral view; L femur in ventral, medial, dorsal, lateral, distal, proximal views. (**f**) L tibia in proximal, ventral views; L astragalus in medial, lateral, distal views; R calcaneum in medial, lateral, distal views; L entocuneiform in medial, lateral, distal views. Skeleton composite scale bar, 5 cm; a-f scale bars, 1 cm.

Here we describe a new partial skeleton of *Mixodectes pungens* (NMMNH P-54501; Fig. 1) recovered from the West Flank of Torreon Wash (locality NMMNH L-6898) within the Ojo Encino Member [*sensu* ^14^], Nacimiento Formation, San Juan Basin, New Mexico (see Supplementary Fig. S1 online). NMMNH L-6898 is stratigraphically located near the middle of the Tj6 fossil horizon [*sensu* ^15^]. The fauna from the Tj6 fossil zone is the primary source among faunas that define the early Paleocene late Torrejonian (To3) NALMA *Mixodectes pungens* interval zone^16^. The age of NMMNH L-6898 is estimated to be 62.4 ± 0.03 Ma based on average sediment accumulation rates between the upper and lower reversal boundaries of a normal polarity zone correlated with Chron C27n^17^. A detrital sanidine age of 62.48 ± 0.02 from within this normal polarity zone from the nearby Escavada Wash provides an additional age constraint for this chron^17^. See^18–20^ for additional information on this locality and other fossil vertebrates collected at this site.

The new dentally associated adult skeleton of *M. pungens* (NMMNH P-54501) is the most complete specimen of a mixodectid yet recovered, preserving a partial skull and much of the axial skeleton, forelimbs, and hind limbs (Fig. 1). Partial disarticulated skeletons of the palaechthonid plesiadapiform *Torrejonia*^18,19^ and the cimolestid *Acmeodon* were recovered from the same horizon, but they are easily distinguished based on their smaller size alone. No repeated tooth loci or skeletal elements have been recognized, which suggests that only one individual of *M. pungens* was present. Previously described fragmentary postcrania attributed to a smaller species, *M. malaris*, were mixed with similar-sized postcrania of the arctocyonid archaic ungulate *Chriacus orthogonius* from the Torrejonian “*Pantolambda* zone” (Tj6 fossil horizon *sensu*^15^) in the San Juan Basin^6^. The new partial skeleton of *M. pungens* analysed here could be distinguished from elements attributed to the smaller *M. malaris*^2,6^ by its larger size. Here, we describe the best-preserved skeletal elements of *M. pungens* and discuss the functional implications of this newly documented morphology. We also compared the new *M. pungens* skeleton to those of other placental mammals including plesiadapiforms, euprimates, treeshrews, and colugos, and we coded it into two morphological data matrices to assess its phylogenetic relationships among eutherian mammals (Crowell et al.^21^ modified from Wible et al.^22^, and Chester et al.^18^ modified from Silcox et al.^23^).

## Results

### Description and functional assessment of NMMNH P-54501

NMMNH P-54501 preserves a dorsoventrally compressed partial skull (Fig. 1A). The rostrum includes a right premaxilla with I1 and a partial crown of I2, a right maxilla with P2-4, M1-2, and roots of M3, a left premaxilla with the root of I2, and a left maxilla with P3-4, M1-2, and roots of M3. A left fragmentary dentary is appressed to the ventral surface of the palate and preserves i1-2, p3-4, and m1-2. The premolar and molar morphology of *Mixodectes pungens* has previously been described^1–3,5^, but NMMNH P-54501 adds previously unknown tooth crowns for this species (I1-2, i2, and P2-3) that confirm *M*. *pungens* has anterior teeth and a dental formula of 2.0.3.3/?2.0.3.3 like its smaller congener, *M*. *malaris*.

The premaxilla is a dorsoventrally tall and robust bone housing a large I1 and a slightly smaller I2 (Fig. 1A). The premaxillary-maxillary suture is caudal to the I2 and thus NMMNH P-54501 confirms the presence of two upper incisors as previously inferred^2^. Both maxillae preserve an infraorbital foramen that is dorsal to the P3 like that of plesiadapiforms, the extant treeshrew *Tupaia*^24^, and the extant colugo *Cynocephalus*^25^, whereas this foramen is often dorsal to a more caudal tooth position in the extant treeshrew *Ptilocercus*^24^ and adapid primates, and dorsal to a more rostral tooth position in omomyid primates^26^.

The rostrum also preserves both nasals, which are displaced ventrally and covered by the maxillae and the paired frontal bones. Due to preservation, it is unclear whether the lacrimal or palatine bones are present in this fragment. Both frontal bones display a caudolaterally projecting triangular postorbital process that arises from prominent anterior temporal lines. In this way, *Mixodectes* differs from non-microsyopid plesiadapiforms, which lack this process, and is more like that of microsyopid plesiadapiforms and extant euarchontans, although treeshrews and primates have much more developed processes that contact the jugal to form a complete postorbital bar^13,24^. The postorbital processes of colugos and microsyopids are triangular but extend far more laterally and are rostrocaudally longer than those of *M. pungens*^27^. The postorbital process of colugos further differs from that of *M. pungens* in that the ventral surface is curved and contours to the shape of the eye^25^. Other isolated bones that are associated with this partial cranium and that we tentatively attribute to *Mixodectes* include a partial left squamosal preserving the glenoid fossa, a partial occipital preserving parts of the nuchal crest and right occipital condyle, and a right petrosal that is too fragmentary to assess beyond the identification of a partial promontorium (Fig. 1).

The postcranial skeleton of *M. pungens* consists of many fragmentary cervical, thoracic, lumbar, sacral, and caudal vertebrae, sternebrae, ribs, and many elements of the forelimbs and hind limbs (Fig. 1). The vertebral column is represented by over forty vertebrae within the expected size range for *M. pungens*. These partial vertebrae and/or vertebral bodies are preserved well enough to attribute to different regions of the spine but only a few can be identified to a specific position (e.g., the axis). The sacrum is crushed but appears to include three vertebrae. The number of caudal vertebrae preserved (approximately 20; Fig. 1) suggests that *M. pungens* had a long tail like that of many arboreal mammals. The ribs of *M. pungens* are narrow (Fig. 1) like those of plesiadapiforms and tupaiid treeshrews, whereas the ribs of *Ptilocercus* and colugos are more craniocaudally expanded, which relates to axial stability^28,29^.

The shoulder girdle is represented by a complete left and a fragmentary right clavicle, the lateral portions of both scapulae, and nearly complete humeri (Fig. 1), which indicate a mobile shoulder like that of arboreal mammals. Both partial scapulae preserve the glenoid fossa, base of the coracoid, and most lateral portion of the scapular spine. The glenoid fossa of *Mixodectes* (Fig. 1C) is concave, almost twice as wide inferiorly as superiorly, and pear-shaped in outline like that of extant euarchontans [e.g., ^30^] and known plesiadapiforms [e.g., ^19^,^31^]. The spherical humeral head of *M. pungens* is greater in diameter than the glenoid fossa and clearly extends superiorly beyond the greater and lesser tuberosities (Fig. 1C), which indicates a mobile glenohumeral joint^30^. The greater and lesser tuberosities are well developed and provide a large area of insertion for the rotator cuff muscles, which, like that of other arboreal mammals, would provide stability to the shoulder joint of *M. pungens* during forelimb abduction^30^. The lesser tuberosity is large and projects medially as in other arboreal euarchontans and would have provided a long lever arm for M. subscapularis, which medially rotates the humerus during vertical climbing^30,32^.

**Fig. 2:**
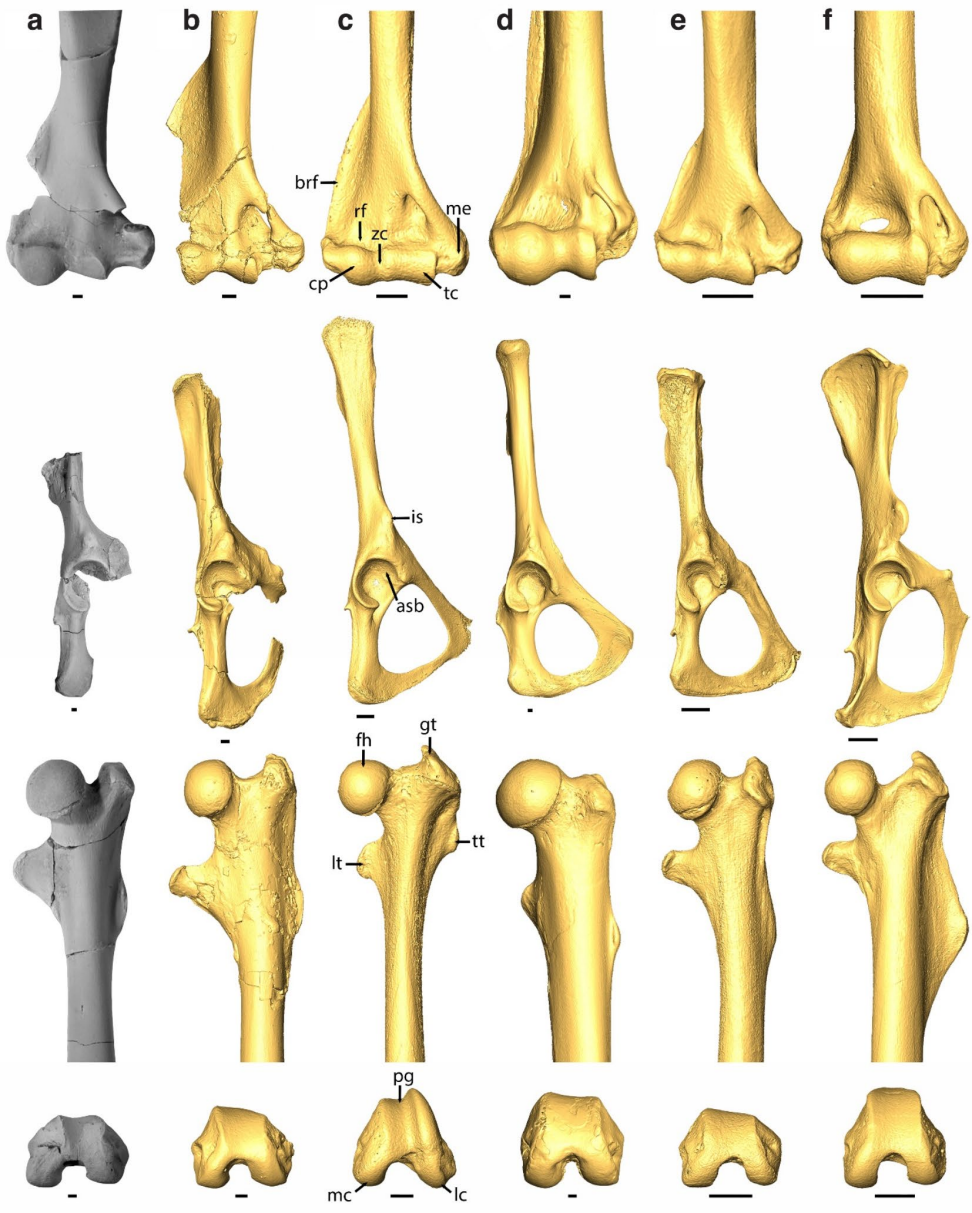
Photographs of (**a**) *Mixodectes pungens* (NMMNH P-54501) postcrania compared to renderings of 3D virtual models based on microCT scan data of (**b**) paromomyid plesiadapiform *Ignacius clarkforkensis* (humerus, UM 108210; innominate and femur, UM 82606), (**c**) euprimate *Galagoides demidoff* (AMNH M-269904), (**d**) colugo *Cynocephalus volans* (ANSP 24797), (**e**) arboreal treeshrew *Ptilocercus lowii* (MCZ 51736), and (**f**) terrestrial treeshrew *Tupaia gracilis* (FMNH 140928). Rows from top to bottom compare right distal humeri in ventral view scaled to width of distal end, right innominates (*Mixodectes* reversed) in lateral view scaled to craniocaudal length of acetabulum, left proximal femora in ventral (above) and distal femora in distal (below) views scaled to width of distal end. Scale bars = 1 mm. Some elements reversed to facilitate comparisons. asb, acetabular cranial articular surface; brf, brachioradialis flange; cp, capitulum; fh, femoral head; gt, greater trochanter; is, anterior inferior iliac spine; lc, lateral condyle; lt, lesser trochanter; mc, medial condyle; me, medial epicondyle; pg, patellar groove; rf, radial fossa; tc, trochlea; tt, third trochanter; zc, zona conoidea. Modified from Chester et al.^19^ figs. 7, 10 and 12. See Supplementary Information for institutional abbreviations.

The elbow joint is represented by both humeri, a left complete and right proximal radius, and both proximal ulnae (Fig. 1), which indicate a mobile elbow, habitual forearm flexion, and capability for manual grasping like that of arboreal mammals that exhibit vertical positional behaviors. *M. pungens* has a large, laterally projecting brachioradialis flange (Figs. 1C and 2A) for the origin of M. brachioradialis and possibly M. brachialis, which contribute to forearm flexion^19^. The humeral radial fossa is excavated for considerable forearm flexion (Fig. 2A), whereas the olecranon fossa is defined but shallow, which would have limited full forearm extension^6^. The radii each have a large bicipital tuberosity near the proximal end (Fig. 1D), which suggests M. biceps brachii was large for forearm flexion and supination that would have assisted in climbing^30^. The humeral capitulum is nearly spherical (Figs. 1C and 2A), and the radial central fossa is circular in outline and excavated (Fig. 1D), both indicating a great ability for forearm supination and pronation^33^; the zona conoidea clearly separates the capitulum and trochlea (Fig. 2A) and would have allowed the radius to rotate more freely in relation to the ulna. The medial epicondyle represents over one third of the distal humeral width (Fig. 2A) and would have provided a large area of origin for the wrist and digital flexors for manual grasping like that of other arboreal euarchontans^30^. As previously reported based on fragmentary postcrania attributed to *M. malaris*, *Mixodectes* distal humeri are like those of plesiadapiforms, *Ptilocercus*, and euprimates, but differ from the derived humeri of colugos, which are much more elongate relative to distal width, have a smaller deltopectoral crest, larger capitulum, narrower medial epicondyle (Fig. 2), and a deeply excavated olecranon fossa for full forearm extension^6^. *Mixodectes* further differs from colugos in having a prominent radial bicipital tuberosity, no evidence of distal fusion between the radius and ulna, a longer olecranon process, a shallower trochlear notch, and no evidence of distal ulnar reduction (Fig. 1D).

The hip joint is represented by both partial innominates and virtually complete femora (Fig. 1), which indicate considerable mobility in the hip and a habitually flexed thigh. The innominate has a craniocaudally elliptical acetabulum and a cranially expanded articular surface (Fig. 2A) as in arboreal euarchontans^32,34^. The elliptical acetabulum allows mobility at the hip joint for wide ranges of abduction and lateral rotation, both important for arboreal climbers, and the cranial buttressing likely reflects loads that were incurred during orthograde positional behaviors on vertical supports^32^. The large and nearly spherical femoral head is slightly taller than the greater trochanter (Figs. 1E and 2A), which would have also contributed to hip mobility. The long, deep trochanteric fossa (Fig. 1E) for the insertion of two obturator and two gemelli muscles would have allowed lateral rotation of the thigh when using vertical supports^19^. The femur has a large, dorsomedially projecting lesser trochanter (Figs. 1E and 2A) providing a large area of insertion for the hip flexor M. iliopsoas^35^. The third trochanter is small (Figs. 1E and 2A) suggesting a less powerful thigh extensor M. gluteus superficialis and a habitually flexed hind limb^34^.

The knee joint was evaluated from the two femora and two broken tibiae, as well as muscle origins from the two partial innominates (Fig. 1), and suggests *Mixodectes* had a habitually flexed knee and was not a specialized leaper or a terrestrial runner. The anterior inferior iliac spine of the innominate is small like that of other arboreal euarchontans (Fig. 2) and would not have provided a large origin for M. rectus femoris for powerful extension of the knee as in specialized terrestrial runners such as tupaiid treeshrews^34^. The femoral condyles are dorsoventrally shallow, and the patellar groove is proximally restricted and shallow (Fig. 2A), suggesting a habitually flexed knee unlike the deeper knees of specialized leapers or terrestrial runners in which M. quadriceps femoris powerfully extends their lower legs^6,34^. Although the anterior inferior iliac spine, femoral condyles, and patellar groove all suggest lack of powerful extension of the knee, the tibial tuberosity is large (Fig. 1F), but this appears to be related to the origin of tibialis anterior; the tibial tuberosity has a prominent lateral protuberance for the origin of M. tibialis anterior, which would have contributed to dorsiflexion and inversion of the foot (see below).

**Fig. 3:**
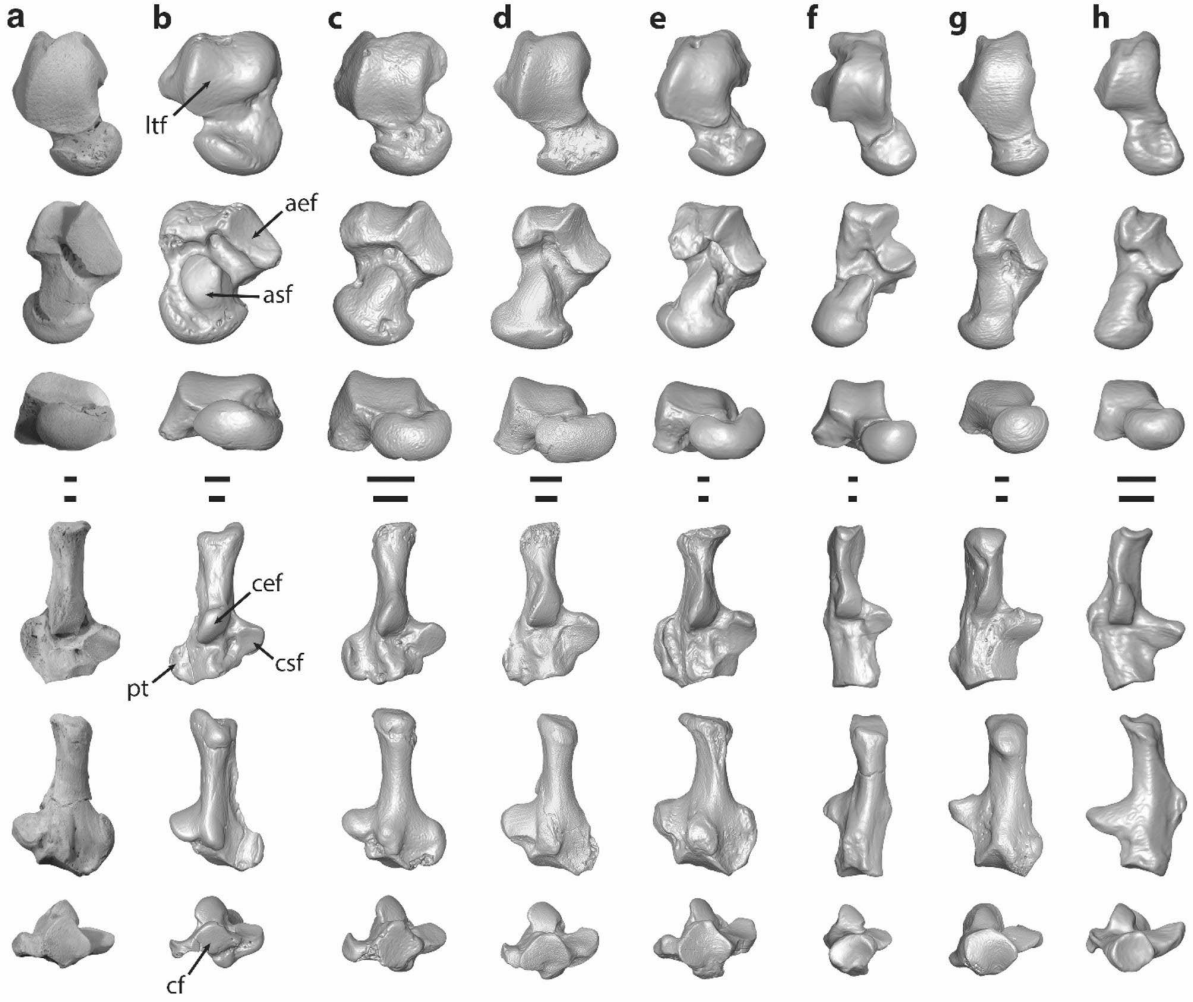
Photographs of (**a**) left astragalus (reversed) and right calcaneum of *Mixodectes pungens* (NMMNH P-54501) compared to renderings of 3D virtual models based on microCT scan data of (**b**) archaic ungulate cf. *Protungulatum* (AMNH FM-118260, FM-118060), (**c**) purgatoriid plesiadapiform cf. *Purgatorius* (UCMP 197509, 197517), (**d**) paromomyid plesiadapiform cf. *Ignacius* (USNM 442235, 442240), (**e**) plesiadapid plesiadapiform *Plesiadapis cookei* (UM 87990), (**f**) adapiform euprimate *Notharctus tenebrosus* (AMNH FM-11474) (**g**) colugo *Cynocephalus volans* (UNSM 15502, AMNH M-207001), and (**h**) arboreal treeshrew *Ptilocercus lowii* (USNM 488072). Right astragali (top three rows) and calcanei (bottom three rows) illustrated in dorsal (top), plantar (middle), and distal (bottom) views. aef, astragalar ectal facet; asf, astragalar sustentacular facet; cef, calcaneal ectal facet; cf, calcaneocuboid facet; csf, calcaneal sustentacular facet; ltf, lateral tibial facet; pt, peroneal tubercle. Specimens scaled to proximodistal length. Scale bars for astragali (top) and calcanei (bottom) all 1 mm. Some elements reversed to facilitate comparisons. Figure modified from Chester et al.^19^ fig. 14. See Supplementary Information for institutional abbreviations.

The upper ankle joint of *Mixodectes* is represented by both partial tibiae and fibulae and a left astragalus (Fig. 1). The distal tibia has a short medial malleolus (Fig. 1F) and an ungrooved articular facet for the lateral tibial facet of the astragalus. The astragalar lateral tibial facet extends far distally onto the neck (Figs. 1F and 3A), which indicates that the foot was habitually dorsiflexed like mammals that cling to vertical supports^36^. The lower ankle joint is represented by the left astragalus and both calcanei (Fig. 1), which possess features such as a confluent sustentacular-navicular facet on the astragalus and a corresponding distal continuation of the sustentacular facet on the calcaneal body like that of extant arboreal euarchontans and plesiadapiforms (Fig. 3), indicating significant mobility for pedal inversion and eversion^36,37^. The calcaneus has a long tuber and a very large, proximodistally long peroneal tubercle like that of plesiadapiforms (Fig. 3), the latter of which would have provided leverage for tendons of the peroneal muscles that contribute to pedal eversion and abduction. The calcaneus is not distally elongated as in leaping euprimates (Fig. 3), a secondary articulation between the posterior side of the sustentaculum tali and the astragalus is present as in some plesiadapiforms and colugos [see ^19^ and references therein], and it appears to have a calcaneal-navicular facet like that of colugos^6,12^. The transverse tarsal joint is represented by the proximal tarsals described above, both naviculars, and a right cuboid (Fig. 1). The calcaneal cuboid facet is subcircular in outline and concave with a plantar pit (Figs. 1F and 3A) that accommodates the convex proximal articular surface of the cuboid, which would have contributed to considerable inversion and eversion of the foot. All these features would have enabled an arboreal animal like *Mixodectes* to navigate uneven and variable branches.

The entocuneiform of *Mixodectes* (Fig. 1F) resembles those reported for non-carpolestid plesiadapiforms in having a mediolaterally wide dorsal side of the distal facet, which suggests *Ptilocercus*-like pedal grasping with a divergent but non-opposable hallux^6,38,39^. The proximal phalanges have pronounced flexor sheath ridges (Fig. 1B), indicating powerful flexion of the digits^35^. Distal phalanges of *M. pungens* (Fig. 1B) are like the previously reported deep distal phalanx^6^ that likely belongs to *Mixodectes*. This deep and mediolaterally narrow ungual morphology is like that of plesiadapiforms and non-euprimate euarchontans [e.g., ^30,35,40^], but the distal ends are not as deep as those of colugos^41^. This combination of features would have allowed *Mixodectes* to vertically cling and climb through trees with its claws.

To estimate body mass of *Mixodectes pungens* (NMMNH P-54501), we measured the maximum femoral length (69.4 mm) and anteroposterior midshaft diameter (5.55 mm) and inputted these values in RStudio using a body weight estimation algorithm (appendix III of Boyer and Gingerich^31^). Body mass was estimated to be 1,370 g (95% CI [375, 4,998]) and 1,270 g (95% CI [668, 2,416]) based on femoral length and midshaft diameter, respectively.

### Phylogenetic analyses

**Fig. 4:**
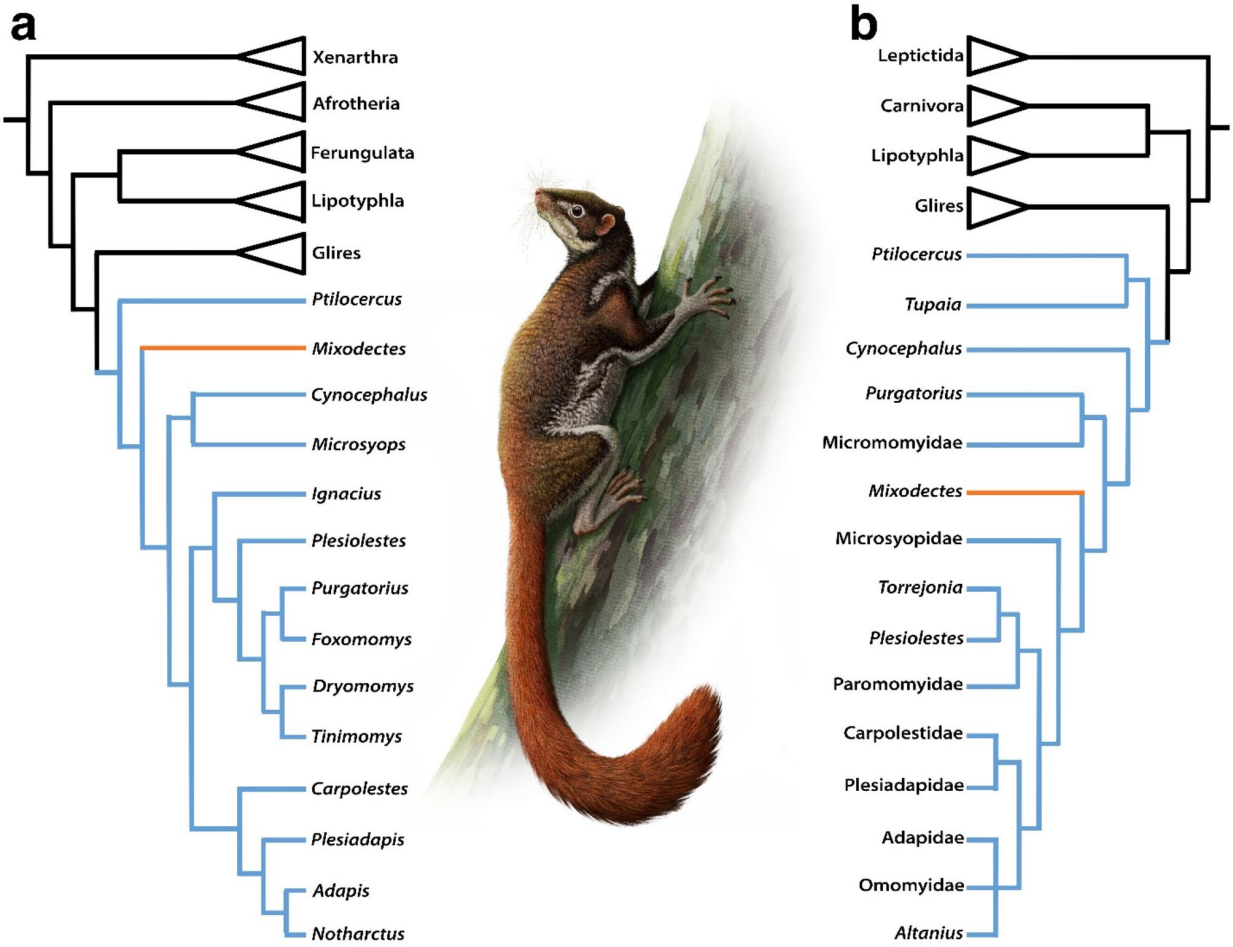
Hypotheses of evolutionary relationships of *Mixodectes pungens* and other eutherian mammals with artist’s reconstruction of *Mixodectes pungens* by Andrey Atuchin. (**a**) Simplified resulting constrained strict consensus cladogram based on modified morphological dataset of Crowell et al.^21^ with Euarchonta indicated in blue and *Mixodectes* supported as a stem primatomorphan and indicated in orange. (**b**) Simplified resulting strict consensus cladogram based on modified morphological dataset of Chester et al.^18^ with Euarchonta indicated in blue and *Mixodectes* supported as a stem primate and indicated in orange. See Supplementary Information for detailed methods, descriptions of morphological characters, specimens examined, and the taxon-character matrices in TNT format.

Unconstrained analysis of the modified Crowell et al.^21^ matrix recovered 200 most parsimonious trees (MPTs) with lengths of 2,925. Strict consensus of these results supports *M. pungens* as the sister taxon to the microsyopid *Microsyops annectens*, and both taxa are supported as the sisters to *Cynocephalus* (see Supplementary Fig. S2 online). Within Euarchonta, Primatomorpha (Primates + Dermoptera) is recovered with the treeshrew *Ptilocercus* as the sister to this grouping. Plesiadapiforms are recovered as either stem primatomorphans (*Purgatorius*, *Foxomomys* *fremdi*, *Tinimomys graybulliensis*, *Dryomomys szalayi*,* Plesiolestes nacimienti*), stem dermopterans (*Microsyops annectens*), or as stem primates (*Ignacius*,* Carpolestes simpsoni*,* Plesiadapis*). The constrained analysis enforced the monophyly of Afrotheria, Xenarthra, Boreoeutheria, Laurasiatheria, and Euarchontoglires, which are generally well-supported in molecular phylogenetic analyses^42^ and recovered 2,976 MPTs with lengths of 2,914. Like the unconstrained strict consensus results, Euarchonta is monophyletic and Primatomorpha is supported. However, in the constrained strict consensus tree, *Microsyops annectens* is recovered as the sister taxon of the extant colugo *Cynocephalus*, *Mixodectes pungens* is supported as a stem primatomorphan, and all non-microsyopid plesiadapiforms are recovered as stem primates (Fig. 4A, Supplementary Fig. S3 online).

Unconstrained analysis of the modified Chester et al.^18^ matrix recovered two MPTs with lengths of 1,021. The strict consensus of these results supports a monophyletic Euarchonta and Primatomorpha. Within Primatomorpha, *M. pungens* is recovered as a stem primate sister to a clade consisting of microsyopids, paromomyoids (Paromomyidae + Palaechthonidae), plesiadapoids (Plesiadapidae + Carpolestidae), and crown primates, with a *Purgatorius* + Micromomyidae clade supported as the most basal primates (Fig. 4B, Supplementary Fig. S4 online). A constrained analysis was not conducted because this morphological matrix recovered clades that are well-supported in molecular phylogenetic analyses.

## Discussion

For over a century, the enigmatic Mixodectidae has been hypothesized to be closely related to members of Euarchonta^1,2,5,10^, but the skeleton of *Mixodectes pungens* described here provides the most compelling evidence for euarchontan affinities to date. This craniodentally associated postcranial skeleton is very similar to that of arboreal euarchontans, including plesiadapiforms, which supports inferences based on less complete material attributed to *M. malaris* that mixodectids are euarchontan mammals^6^. Mixodectids have long been considered a possible ancestral group to extant colugos based on several features of their dilambdodont dentition (e.g., large upper molar conules, crest-like lower molar paracristid), but this hypothesis has been challenged more recently given that some of these similarities appear to evolve within the genus *Eudaemonema*, suggesting they evolved independently from colugos (e.g.,^7^). Similarly, dental features have long linked Mixodectidae to the contemporary plagiomenids, which in turn were considered extinct dermopterans, but more recent non-dental discoveries of plagiomenids including a partial skull^9^ and postcrania^43^ suggest they are neither dermopterans nor euarchontan mammals. Another extinct family that may represent the closest known relatives of mixodectids based on dental similarities is the Microsyopidae, but comparisons are limited because dentally associated postcrania of microsyopids have not yet been reported.

All results from phylogenetic analyses incorporating novel data from NMMNH P-54501 support *Mixodectes* as a primatomorphan mammal (Fig. 4). Although differences among resulting topologies presented here are clearly due to character and taxon sampling, and because one matrix was not designed (nor were characters modified) to test interrelationships among plesiadapiforms^21^, all three hypotheses of mixodectid relationships are worth considering. The unconstrained results of the modified Crowell et al.^21^ matrix support *M. pungens* and the microsyopid *M. annectens* as sister taxa and as stem dermopterans, whereas the constrained results support *M. annectens* as the sister taxon to the extant colugo *Cynocephalus,* and *Mixodectes* as a stem primatomorphan (Fig. 4A, Supplementary Figs. S2-3 online). The unconstrained analysis of the modified Chester et al.^18^ matrix supports *Mixodectes* as a stem primate sister to a clade of microsyopids, paromomyoids, plesiadapoids, and euprimates (Fig. 4B, Supplementary Fig. S4 online). *Mixodectes* postcrania are very similar to those of plesiadapiforms, but many of these similarities are likely retained euarchontan synapomorphies and therefore not informative for evaluating the alternative tree topologies presented here. If mixodectids are stem dermopterans, some of the proposed dental (e.g., dilambdodonty) and postcranial (e.g., calcaneonavicular facet) similarities between *Mixodectes* and colugos might be synapomorphies or parallelisms even though colugo-like specializations for folivory, suspension, and gliding are not present in *Mixodectes*. If mixodectids and plesiadapiforms are stem primatomorphans or stem primates, the dental specializations of mixodectids are likely autapomorphic. Also, mixodectids and microsyopids may be more closely related to each other than to non-microsyopid plesiadapiforms (as supported by the unconstrained results of the modified Crowell et al.^21^ matrix; Supplementary Fig. S2 online), but we question phylogenetic results that support microsyopids nested among non-microsyopid plesiadapiforms (e.g., Fig. 4B) given that they are distinct from other plesiadapiforms in their dental and inferred bullar morphology^2,21,26,27^. Again, regardless of which phylogenetic hypothesis is correct, all analyses conducted here support *Mixodectes* as a primatomorphan.

Postcranial evidence indicates that *Mixodectes pungens* was a claw climbing arborealist that often clinged to vertical supports like tree trunks. Body mass of NMMNH P-54501 based on femoral length and midshaft diameter is estimated to be 1,370 g and 1,270 g, respectively, which confirms that *M. pungens* was a very large primatomorphan mammal for the early Paleocene of North America^44^. Based on estimated body mass and molar features, such as the presence of a hypocone and strong wear on crests^2^, *M. pungens* was omnivorous and likely consumed leaves but was not as specialized for shearing as extant^25^ or extinct^45^ colugos. Regardless of whether mixodectids are stem dermopterans, which is further complicated by these clades being geographically restricted to western North America and southern Asia, respectively, *Mixodectes* serves as a model for colugo origins as an arboreal primatomorphan that increased body mass dramatically and started moving away from a more frugivorous diet like that common among many plesiadapiforms. In this way, mixodectids appear to have exploited a new ecological niche in North America, potentially driven by sympatric plesiadapiforms such as *Torrejonia wilsoni*, also documented at this locality, which were also arboreal but smaller bodied and more frugivorous. Therefore, new insights from this skeleton of *Mixodectes* add to understanding not only where this enigmatic family fits in the tree of life but also how Euarchonta and Placentalia diversified ecologically in the early Cenozoic following the Cretaceous-Paleogene mass extinction.

## Methods

### Phylogenetic analyses

The new skeleton of *M. pungens* was coded into two complementary character-taxon matrices to assess relationships among eutherian and euarchontogliran mammals, respectively (see Supplementary Information). The first matrix was modified from Crowell et al.^21^, which was originally designed to test relationships among Cretaceous eutherians and crown placental mammals and provides a broad selection of eutherian mammals and rigorous character sampling^22^. The second matrix was modified from Chester et al.^18^, which was originally designed to test interrelationships within Euarchontoglires^23^. Together, these matrices provide independent tests of the phylogenetic position of *Mixodectes pungens* within Eutheria and, more specifically, among euarchontogliran mammals. Cladistic analyses using maximum parsimony were conducted in TNT (v. 1.5)^46^. Multistate characters were unordered and New Technology Search was executed using 100 replications as the starting point for each hit, 200 iterations of ratcheting, 10 rounds of tree drifting, 10 rounds of tree fusing, and sectorial searching. The resulting MPTs were used as starting trees in a Traditional Heuristic Search that was executed using tree-bisection and reconnection.

## Electronic supplementary material

Below is the link to the electronic supplementary material.

Supplementary Material 1

## Data Availability

The datasets generated and/or analysed during the current study are available in the Morphobank.org repository, http://morphobank.org/permalink/?P5501. See Supplementary Information for additional information.
