# Supplementary material for: New remarkably complete skeleton of Mixodectes reveals arboreality in a large Paleocene primatomorphan mammal following the Cretaceous-Paleogene mass extinction
Source: Sci Rep. 2025 Mar 11;15:8041. doi: 10.1038/s41598-025-90203-z (PMC11897203; doi:10.1038/s41598-025-90203-z)
Supplement: Supplementary file 1 — Supplementary Material 1 [file 41598_2025_90203_MOESM1_ESM.docx]

**Scientific Reports**

**Supplementary Information**

**New remarkably complete skeleton of *Mixodectes* reveals arboreality in a large Paleocene primatomorphan mammal following the Cretaceous-Paleogene mass extinction**

Stephen G. B. Chester^1,2,3*^, Thomas E. Williamson^4^, Jordan W. Crowell^2,3^, Mary T. Silcox^5^, Jonathan I. Bloch^6^, Eric J. Sargis^7,8,9^

^1^Department of Anthropology, Brooklyn College, City University of New York, 2900 Bedford Avenue, Brooklyn, NY, 11210, U.S.A.

^2^PhD Program in Anthropology, The Graduate Center, City University of New York, 365 Fifth Avenue, New York, NY, 10016, U.S.A.

^3^New York Consortium in Evolutionary Primatology, New York, NY, 10024, U.S.A.

^4^New Mexico Museum of Natural History and Science, 1801 Mountain Road, NW, Albuquerque, NM 87104-1375, U.S.A.

^5^Department of Anthropology, University of Toronto Scarborough, 1265 Military Trail, Scarborough, ON, M1C 1A4, Canada.

^6^Florida Museum of Natural History, University of Florida, 1659 Museum Road, Gainesville, FL, 32611-7800, U.S.A.

^7^Department of Anthropology, Yale University, P. O. Box 208277, New Haven, CT, 06520

^8^Divisions of Vertebrate Paleontology and Vertebrate Zoology, Yale Peabody Museum, New Haven, CT, 06520, U.S.A.

^9^Yale Institute for Biospheric Studies, New Haven, CT, 06520, U.S.A.

Correspondence to: Stephen G. B. Chester. Email: [stephenchester@brooklyn.cuny.edu](mailto:stephenchester@brooklyn.cuny.edu)

This PDF File Includes:
S1. Geological setting and age of locality NMMNH L-6898 2

S2. Materials 4

Anatomical and Institutional Abbreviations 4

Specimens examined 5

S3. Phylogenetic analysis 6

Character-taxon matrix modified from that of Crowell et al. (2024) 6

Character-state revisions 25

Force commands 27

Cladistic methodology and results 28

Resulting synapomorphies 30

Character-taxon matrix modified from that of Chester et al. (2017) 35

Character-state revisions 40

Cladistic methodology and results 42

Resulting synapomorphies 43

Figures S1-S4 45

References 53

**S1. Geological setting and age of locality NMMNH L-6898**

Geochronology

The new partial skeleton of *Mixodectes pungens* (NMMNH P-54501) analyzed here was collected at locality L-6898 from the West Flank of Torreon Wash and occurs stratigraphically from near the middle of the Tj6 fossil horizon (*sensu* [1]) within the Ojo Encino Member (*sensu* [2]), Nacimiento Formation (Fig. S1). The fauna from the Tj6 fossil zone is the primary source among faunas that define the late Torrejonian (To3) North American Land Mammal Age (NALMA) *Mixodectes pungens* interval zone [3]. The age of locality L-6898 is estimated to be 62.4 ± 0.03 Ma based on average sediment accumulation rates between the upper and lower reversal boundaries of a normal polarity zone correlated with Chron C27n (locality # 25, table 1 and fig. 3 in [4]). A detrital sanidine age of 62.48 ± 0.02 Ma from within this normal polarity zone from nearby Escavada Wash provides an additional age constraint for this chron [4].

Paleoenvironmental reconstruction

The strata contained within Tj6 are interpreted to be distal floodplain deposits on a fluvial fan that prograded from northwest to southeast across the San Juan Basin through the early Paleocene [5]. Locality L-6898 is within a mud to very fine sand that lacks obvious pedogenic alteration and likely represents waning flood deposits that accumulated as a distal portion of a crevasse splay on a distal floodplain. This interpretation is supported by the presence of articulated and semi-articulated skeletons of small vertebrates at L-6898 that probably would not have maintained close association if they had been transported a significant distance.

Paleocene strata of the San Juan Basin preserve an excellent floral record. Megafossil floras collected from the Ojo Encino Member, Nacimiento Formation of Torreon Wash, are dominated by dicot angiosperms, with minor contributions of Pteridophytes, Monocots, and other plants [6]. Common middle to late Torrejonian plants from the Nacimiento Formation include *Platanites raynoldsi* (sycamore), *Juglandiphylloites glabra* (walnut/pecan), *Aesculus hickeyi* (Sapindales), *Macginitea nobilis* (lobed sycamore), Laural relatives, *Equisitum* (horsetails), and palms. Conifers are absent (Flynn, personal comm.).

Mean annual temperature (MAT) and mean annual precipitation (MAP) estimates based on Leaf Margin Analysis of collections of fossil leaves from the Ojo Encino Member reconstruct the biome of sampled plant communities to be within modern temperate forest to rainforest to seasonal tropical forest [6]. These communities experienced mesic conditions, probably with seasonal variation in precipitation [6].

**S2. Materials**

Anatomical Abbreviations

I, upper incisor; i, lower incisor; P, upper premolar; p, lower premolar; M, upper molar; m, lower molar; L, left; R, right.

Institutional Abbreviations

AMNH, American Museum of Natural History, New York, New York, U.S.A.; ANSP, Academy of Natural Sciences of Philadelphia, Drexel University, Philadelphia, Pennsylvania, U.S.A.; DLC, Duke Lemur Center, Durham, North Carolina, U.S.A.; FMNH, Field Museum of Natural History, Chicago, Illinois, U.S.A.; KUVP, Kansas University, Vertebrate Paleontology, Museum of Natural History, Lawrence, U.S.A.; MCZ, Museum of Comparative Zoology, Harvard University, Cambridge, Massachusetts, U.S.A.; NMMNH, New Mexico Museum of Natural History and Science, Albuquerque, New Mexico, U.S.A.; UCMP, University of California Museum of Paleontology, Berkeley, California, U.S.A.; UM, University of Michigan Museum of Paleontology, Ann Arbor, Michigan, U.S.A.; UNSM, University of Nebraska State Museum, Lincoln, Nebraska, U.S.A.; USNM, United States National Museum of Natural History, Smithsonian Institution, Washington D.C., U.S.A.; UW, University of Wyoming, Laramie, Wyoming, U.S.A.

Specimens Examined

Fossil Specimens: Archaic ungulates cf. *Protungulatum* (AMNH FM-118260, 118060), *Chriacus orthogonius* (NMMNH P-19995), cimolestid *Acmeodon secans* (NMMNH P-54499); mixodectid *Mixodectes pungens* (AMNH FM-2557b, 3081; NMMNH P-54501), *Mixodectes malaris* (AMNH FM-3080; NMMNH P-3088), purgatoriid plesiadapiform cf. *Purgatorius* (UCMP 197509, 197517), micromomyid plesiadapiforms *D. szalayi* (UM 41870) and cf. *Tinimomys graybulliensis* (USNM 442277, 442280), microsyopid plesiadapiform *Microsyops annectens* (UW 12362), palaechthonid plesiadapiforms *Plesiolestes nacimienti* (KUVP 9557) and *Torrejonia wilsoni* (NMMNH P-54500), paromomyid plesiadapiforms *Ignacius clarkforkensis* (UM 82606, 108210), cf. *Ignacius* (USNM 442235, 442240), *Ignacius graybullianus* (USNM 421608), and cf. *Phenacolemur simonsi* (USNM 442260), plesiadapid plesiadapiforms *Nannodectes gidleyi* (AMNH FM-17379) and *Plesiadapis cookei* (UM 87990), carpolestid plesiadapiform *Carpolestes simpsoni* (UM 101963, USNM 482354), and adapiform euprimate *Notharctus tenebrosus* (AMNH FM-11474).

Modern Specimens: euprimates *Microcebus murinus* (USNM 83656, 83657), *Galagoides demidoff* (AMNH M-269904), and *Galago moholi* (DLC 2016f), colugo *Cynocephalus volans* (AMNH M-207001, ANSP 24797, FMNH 56442, UNSM 15502), and treeshrews *Ptilocercus lowii* (MCZ 51736, USNM 488072), *Tupaia gracilis* (FMNH 140928), and *Tupaia minor* (FMNH 141464).

**S3. Phylogenetic Analysis**

Character-taxon matrix modified from that of Crowell et al. (2024)

Modified character-taxon matrix of [7] (originally derived from [8] and subsequently modified by [7,9-13]). This modified character-taxon matrix (with the additional taxon, *Mixodectes pungens*, in bold) is provided below in TNT format and is freely available in the Morphobank.org repository, <http://morphobank.org/permalink/?P5501>. The character and character state definitions are provided in [9,11] and are also freely available on Morphobank.

*Nanolestes* spp. 000?0??0??????0?1?0?000000??00000?00000001000?000000000000100?0000?01020110[0 1]00000000??000??0????????0?0??000000001000010?0???0?0020?0001????0020000?1?02000?00?????????????????????????????????????????????????????????????????????????????????????????????????????????????????????????????????????????????????????????????????????????????????????????????????????????????????????????????????????????????????????????????????

*Peramus tenuirostris*

000[0 3]0??[0 1 2]??????????0?000101??0?100000000001000000000000110001100000000200000101000000??000??0????????0?00?00000000100000100???0002[1 2]01011110?100200010?10100001????????0?????????????????????????????????????????????????????????????????????????????????????????????????????????????????????????????????????????????????????????????????????????????????????????????????????????????????????????????????????????????????????????

*Vincelestes neuquenianus*

003000110000000000000000010?300????00000000001?????00000??0010100001020010022101100100000?01000000??0211001000000300100100???01032110011201100001011010201?011000000[0 1]00000000?0000100000000000000000000000000000000000000000?00000000000000001?00?000000?00000000??0000000000000?00000000000000000000000000000000000?0000000??0?000000010000000000000?0200?0000000?0000000000000000000000?00?00000?00000000000000100000000?????

*Kielantherium gobiensis*

000???????????????0?????????1?????????????????000?????????0?000111011200022[0 1]1100000111100?02000000??0?1??02000000110010100???01[0 1]?101?????01??????????????010???????????????????????????????????????????????????????????????????????????????????????????????????????????????????????????????????????????????????????????????????????????????????????????????????????????????????????????????????????????????????????????????????

*Deltatheridium pretrituberculare*

00100011000000000?0?01010101210010?00000000000101??00000000001011101000002211100101011122012000000??01110021000001100101110?001013010011100001100010110001?1111100001000?0010?00100000100?0000001?010000000000??10????????01??????????1121?????????????????1?[0 1]01100????1???10[0 2]?0?20111?0202110000?0??0002?2000001000?0101???????1?0??012???01?0?0????????????????????????????????????????????????????????????0000??00??0???????

*Mayulestes ferox*

001000000000000000000101010?2?0100?00000000000100???0000000001011101002012212210101111122012110000??00110001101011130113110001001301?0???0????????????00011??1111011100001010?0010000010100011101000000000000?000000?0010?00??0?11000011110010100?011110?1010011100?0001000101?1?201110011?10000011100002?2100001000?0111000??0?1000?0?????????201100100???????1100111000110000???1000110010?00000?0100110?0010001100000???????

*Pucadelphys andinus*

001000000000000000000101010?2?0100?00000000000101??00000000001011101002012112210110111122022110100??001100001011111301231200010013010021100011100011110001?1111100011000000110001000001010???1111000000000000000000001010?00?2011000?01120??1?100?111110?101001110010001000101?1?201110010210000010100002?2100000000?0111000??0?1000?0120?01???2011000000100???110010100011000???00000110000?0000??0?0?1100001000110000002111??

*Acristatherium yanensis*

000[0 1]00110000020410001001010?0?10010000000000000010000000000011111101100000001100000000100?12000000??0?1??00000100?1?0111?100000???010001?01????????001??0?????0000011010?001001001100??????????????01000010?0???????1?02?00??????01100?1[0 1]?????????????????0001?1????????????????????????????????????????????????????????????????????????????????01?????????????????????????????????????????????????????????????????????????????

*Eomaia scansoria*

0001000000000?000?000001010?0?100101000101?????010010010?00?100[0 2]10?1?0?002?[0 1]0100?00??1111012??00?0??001?1010?010?01?02??01?000002101011120110020001001020010101100011??0??010?100?1??0?????????????0?00001???????????????????????0?0?0?????????????????????????1????0??????????????11?????????????????????????????????????????????????????????????????????0????00?0?00???110?000??0???1?0?111?0???????11?0??0??10?00???????????

*Prokennalestes* spp.

000?00?0??????????0?0???00??0?1????10001[0 1]1000000000[0 1]00[0 1]000[0 1]0100[1 2]1001100010200100000011111012000000??001000000010011002111100000011010111201100200010?100001010????011?1??????????????????????????????0?0??????????????????????????????11[0 1]??????????????????????????????????10000?00111?0001110000??11000010000000?00?000????????1?00??02???11?0????????????????????????????????????????????????????????????????????????????????

*Murtoilestes abramovi*

00????????????????????????????????????????????????????????????0111?2100010211100000011112012000000??0?1??000101?01110211110000?????????????????????????????????????????????????????????????????????????????????????????????????????????????????????????????????????????????????????????????????????????????????????????????????????????????????????????????????????????????????????????????????????????????????????????????????

*Bobolestes zenge*

000?00?0??????0???0?0???11??0?1???????????????00000000120000100110?20010022[0 1]1100100111122112010100??00100000001001110211110000002[1 2]01??01?01???????????01000021?????????????????????????????????????????????????????????????????????????????????????????????????????????????????????????????????????????????????????????????????????????????????????????????????????????????????????????????????????????????????????????????????

*Montanalestes keebleri*

00[0 1]????[0 1 2]????????????????????[0 1]?1????????????????????100110001110??????????????????????????????????????????01000100111021111000000[1 2]3010021201100201010110[0 2]01?021?????????????????????????????????????????????????????????????????????????????????????????????????????????????????????????????????????????????????????????????????????????????????????????????????????????????????????????????????????????????????????????????????

*Sheikhdzheilia rezvyii*

00?????????????????????????????????????[1 2]??????????????1010?110012101020002101111102101122122010200?00?1??000?010011[1 2]1223?20000?[0 1]?20????????????????????????????????????????????????????????????????????????????????????????????????????????????????????????????????????????????????????????????????????????????????????????????????????????????????????????????????????????????????????????????????????????????????????????????

*Alostera saskatchewanensis*

00????????????????????????????????????????????????????????????0121?2021002222111102111122122110222000?1??0?0?01?01131223?20000?????????????????????????????????????????????????????????????????????????????????????????????????????????????????????????????????????????????????????????????????????????????????????????????????????????????????????????????????????????????????????????????????????????????????????????????????

*Lainodon orueetxebarriai*

00????????????????????????????????????????????????????????????0??????????????????????????????????????????001001001131223?20000?????????????????????????????????????????????????????????????????????????????????????????????????????????????????????????????????????????????????????????????????????????????????????????????????????????????????????????????????????????????????????????????????????????????????????????????????

*Avitotherium utahensis*

00????????????????0?????01??1??????????????????00??000????????0121?20210022[0 2]2111102111122122010[1 2]11000?1??001002100131221?20001?????????????????????????????????????????????????????????????????????????????????????????????????????????????????????????????????????????????????????????????????????????????????????????????????????????????????????????????????????????????????????????????????????????????????????????????????

*Gallolestes* spp.

00??????????????????????????????????????????????????0012?????10????????????????????????221?2110?1[1 2]00??1??0000021001302231200010??2?????????????????????????????????????????????????????????????????????????????????????????????????????????????????????????????????????????????????????????????????????????????????????????????????????????????????????????????????????????????????????????????????????????????????????????????

*Parazhelestes* spp.

000???????????0?0?00????0[0 1]0?0?1????1000111000010010010021?0?100122121210022221111021111221221112220000100000002111121223020001001201???1201??????????????1?121?????????????????????????????????????????????????????????????????????????????????????????????????????????????10[1 2]?0?201?1??002100100??????0[0 1]?0?0000?000??[1 2]???????????00??020?111?0?0??????????????????????????????????????????????????????????????????????????????

*Aspanlestes aptap*

000?0??[0 1 2]????????????????????0?1?1?01??01111100100000100210011002221212100220211110211112212211022200001??000?0111112122302000100[1 2]101??11201??????????????1?121?????????????????????????????????????????????????????????????????????????????????????????????????????????????10[1 2]?0?201?1??002100100??????0[0 1]?0?0000?000??[1 2]???????????00??020?111?0?0??????????????????????????????????????????????????????????????????????????????

*Zhelestes temirkaysk*

00010?????????????????01010?0?1?101100011111?010010010021???10022212121002[1 2]221111021[0 1]11???22110[1 2][1 2]2000010000000111112122302000100120?0?1120?10020001001???1?121?????11??????????????????????????????????????????????????????????????????????????????????????????????????????10[1 2]?0?201?1??002100100??????0[0 1]?0?0000?000??[1 2]???????????00??020?111?0?0???????????????????????????????????????????????????????????020110011100??????0

*Paranyctoides* spp.

000?0?????????????0?????11??0?1????1[0 1]001[1 2]2000?0001010000[0 1]001100111011010[0 2]22[0 1]1111100111112112111111000?1??00000100113122112000100[1 3]201??11?0???????????????1?1???????????????????????????????????????????????????????????????????????????????????????????????????????????????????????????????????????????????????????????????????????????????????????????????????????????????????????????????????????????????????????????????????

*Eozhelestes mangit*

000????0??????????0?????11??0?????????????????000[0 1]?1????????1?0??????????????????????????????????????????000001001110223020001?0?2????????????????????0[0 1]0??????????????????????????????????????????????????????????????????????????????????????????????????????????????????????????????????????????????????????????????????????????????????????????????????????????????????????????????????????????????????????????????????????

*Cimolestes* spp.

001[0 3]00?1??????021?001001010?1?1?10?10012[0 1][0 1]0000010??000000000100110010210022001011001111221120101[0 1][0 1]0000100000000001110211120[0 1]00001[1 2]01001120010????000?00[0 1]01?121????11[0 1]?10??0????01?110???????2010??01???????????????????10?????????????????????????????????????????????????????????????????????????????????????????????????????????????????????????????????????????????001???????????????????????????????????????????????00000??

*Maelestes gobiensis*

000[0 3]00?1??????0?100?1?0[0 1]010?0?10101100110211000111100000001010022212021002100101100101100?1201011100001000000021111102121210000023010021201100200001020001?1???11001100???0???10??1000????1102101100100001110110000001?[0 1]000????0?11???1111?0112120???[2 3]???1100?011001000300010000?01111002021100000001001[1 2]?[1 2]00100011111[1 2]00[0 1]100?0?00??10021111110[0 3]01100010???????000?1000011100??????????????????01100???11??0???????????????????

*Batodon tenuis*

001???????????????0?????01??1?1????100?112000?010??000110001100221?102100212110110011112211201011100001000000010111[1 2]02121[1 2]00000022?1??????????????????0001?????????????????????????????????????????????????????????????????????????????????????????????????????????????????????????????????????????????????????????????????????????????????????????????????????????????????????????????????????????????????????????????????????

*Bulaklestes kezbe*

001???????????????0?0???00??1?1????1000???????000??000100001100112111210022[0 2][0 1]101100111112112001100??001?0000?010011102112100000002?1??????1???????????0001??21?????????????????????????????????????????????????????????????????????????????????????????????????????????????????????????????????????????????????????????????????????????????????????????????????????????????????????????????????????????????????????????????????

*Daulestes* spp.

001???????????????0?????00??1?1????10001120000000??000101011100111[0 1]10000022001011001111121120001[0 1][0 1]000?1??000?02001110211?10000?0?2????????1??????????????1?????????1???????????????????????????????????????????????????????????????????????????????????????????????????????????????????????????????????????????????????????????????????????????????????????????????????????????????????????????????????????????????????????????

*Uchkudukodon nessovi*

001[0 3]00?[0 1]??????[0 1]???0?000?0000101????10001020000??0??000?0101?100110111000022001011001111121120001[0 1][0 1]000?100000002001110211210000001[2 3]010111201100200010010201?121??0?011000?1110?100111000???0[0 1]0210110?1000011001[0 1 3]?00000?02??010?00210???1111???1?00?021[2 3]00?1?00?0111012?1?0001000000011100?1?1?0000??????1??????00????????????00?0?000????????????0?100??????????????????????????????????????????????????????????????????????????

*Kennalestes gobiensis*

001000[0 1 2]1?00001000?0?00000000101000?10011121100001??000001010100212021200022001011001111121120011120000100000001011110211110000001201002120010020000??10001?1211100011010?0?10?101111000???0000101100?0000110?10000000??2???10100???0001111?01?2?20?11300?1000?01110121?3000101?000111100?1?10000000?1?11??[0 1]00100211101100110?1?00000?00211112??20?110??????????????????????????????????????????????????????????????????????????

*Asioryctes nemegtensis*

00100000000001000?000000000?1?1000?20011220000001??1001010101002100102100220010110011111[0 2]112001100??001000000020111102111100000001010021200100200010010001?1211100011010?0010?1001110000000??010110000010110?10000000??2??010100[0 2]100001111?0111020011300?1000101110121?3000101?00011110001?10000000?1111??[0 1]0010021110110011001?00000?0??????2??10111001101???????????????????000??????????????0011?0?11111?00??1000000?0???????

*Ukhaatherium nessovi*

001000000000010000000001010?1?1001?20011220000001??10010101010021001021002200101100111110112001100??001000000020?11102111100000002010021200100200010010001??21110001101000010?1011110000000??01011000001011?01?0??00??02??01010??1?0001111?01110200?1?00?100010111012103000101?000111100?1?10000000?1?11[0 1]1[0 1]001002111?11000100110?0001002???12??2011????????????100010000011?00?0?00??01?00?11?0011001112100001010200?220????0?0

*Deccanolestes hislopi*

001?0[0 1]??????????????????011?1?0???010011030000001001011211001101110100000220[0 1]1[0 1]010011002[0 2]11211[1 2][0 1]00??00100000[0 1]00[0 1]011111[1 2]1211000001101011???????????????????????????????????????????????????????????????????????????????????????????????????????????????????????????????????????????????????????????????????????????????????????????????????????????????????????????????00011????????????????????02200110111000201101111011110111

*Deccanolestes* cf. *D. hislopi*

00????????????????????????????????????????????????????????????0[1 2][1 2][1 2]0[0 1]00000220[0 1]11010011002[0 2]1[1 2]2111[0 1]00??001000000001011111112110000????????????????????????????????????????????????????????????????????????????????????????????????????????????????????????????????????????????????????????????????????????????????????????????????????????????????????????????????????????????????????????????????????????????????????????????????

*Deccanolestes robustus*

00????????????????????????????????????????????????????????????011100010002201110100110012012112[0 1]00??0?1??0000000011111112110000????????????????????????????????????????????????????????????????????????????????????????????????????????????????????????????????????????????????????????????????????????????????????????????????????????????????????????????????????????????????????????????????????????????????????????????????

*Deccanolestes narmadensis*

00???????????????????????????????????????????????????????????????????????????????????????????????????????0[0 1][0 1]00[0 1][0 1]011111[1 2]12110000????????????????????????????????????????????????????????????????????????????????????????????????????????????????????????????????????????????????????????????????????????????????????????????????????????????????????????????????????????????????????????????????????????????????????????????????

*Kharmerungulatum vanvaleni*

00???????????????????????????????????????????????????????????????????????2?????????????????????????????????????00?1?1?11?1?000?????????????????????????????????????????????????????????????????????????????????????????????????????????????????????????????????????????????????????????????????????????????????????????????????????????????????????????????????????????????????????????????????????????????????????????????????

*Afrodon germanicus*

00????????????????????????????????????????????????????1211????021[1 2]010000021011101001100221[1 2]2111[0 1]00??001??000000001111121?21000??????????????????????????????????????????????????????????????????????????????????????????????????????????????????????????????????????????????????????????????????????????????????????????????????????????????????????????????????????0?00011???????????0?????????2200100?100002011111110111111?1

*Afrodon chleuhi*

00????????????????????????????????????????????????????1210????0[1 2]1[0 1]0[1 2]00000220111010011002212211[0 1][0 1]00??001??0[0 1]100[0 1]0011111[1 2]12[1 2]1000?????????????????????????????????????????????????????????????????????????????????????????????????????????????????????????????????????????????????????????????????????????????????????????????????????????????????????????????????????????????????????????????????????????????????????????????????

*Kulbeckia kulbecke*

001100[0 1 2]0?????21?1?11100000??1?1000?2?111020000000???001211?110022002021002221111102111112122001200??001?00000022111[1 2]122211000000[1 2]101???1210???????????0001?12111000110000?0?0??00????101???[0 1 2]??????0010000????????????1?10?????????????1????????????????????????????????????10[1 2]?0?0[0 1]1?1?011?110000????1?011[0 1]00100?111?11???????????00???2???11?0?0??????????????????????????????????????????????????????????????????????????????

*Zhangolestes jiliensis*

000?00?1??????1?1?211???111?0?????????????????00011???121??0100??????????????????????????????????????????000001?01131222??0000?[0 1]?101??????????????????0?0??????????????????????????????????????????????????????????????????????????????????????????????????????????????????????????????????????????????????????????????????????????????????????????????????????????????????????????????????????????????????????????????????????

*Alymlestes kielanae*

00????????????????????????????????????????????????????????????0??????????????????????????????????????????001002210131223???000?????????????????????????????????????????????????????????????????????????????????????????????????????????????????????????????????????????????????????????????????????????????????????????????????????????????????????????????????????????????????????????????????????????????????????????????????

*Zalambdalestes lechei*

00[1 2][0 1]00[2 3]11110?21211211000111?[1 2]?1000?21111120001000??00012100010022002021002121111102101100?22001200??00110001002211131223121000001101011120[0 1]100200001010001?121110001100000110?1000100101000200[0 1]011010000011101200110100000010100101[0 1]00111100111120011300?1000[1 2]0111012103000101?00011110011?100000000101111[0 1]00100211111[1 2]0000001101000?102???1100101???00?11?????11??100001???0?????01???100??0??[1 2]2200?1?110[1 2]01201?30112?000000?0

*Barunlestes butleri*

002[0 1]00?1??????1211211011111?2?1????201111200?000???0??12100010022002021002121111102101100?22001200??0011000100221113122312100001?1010111200100200001010101?121???0?11000????0?100?110?0???0220101001?00????1?1[1 3]01100??0????10?0??????011?1??11112001130??1000?0111012103000101?0?011110011?10000000?1?11???00100211111?00000????1?0??1??????????011100?0???0?0?1???100001110?00100012111001?0??????????????????????????????????

*Gypsonictops* spp.

00[0 1]?00?[0 1 2]??????????0?0???010?[0 1]?1????2[0 1]11222111000010[0 1]1[1 2]121101100221010210022211111001111221220112220000100000002[1 2]111312[1 2]1120000002201???1200??020000??????1?121?????11??????????????????????????????????????????????????????????????????????????????????????????????????????????????????????????????????????????????????????????????????????????????????????????????????????????????????????????????????????????????????????????

*Leptictis* spp.

001300311000000000000001110?101001?11112221110010??000121201100220020210022211111021111221120112120000110000002[1 2]111312211200000012010111200100200001120?01?1211100112111011110101011001000002011100000010101100000000102??0110001001001111111020101212010110020110011001100100000011110011?10000001?10001?1001002100?111110000101100100211012??301?111????000201111000001110?00010100111002?00102200010210200201011112100000000

*Purgatorius* spp.

001?00?1??300?0?1?0?1???11??1??????????2[1 2]11100010??00020010110022[0 1]02021002222[1 2]11102110112112112222000?1??001001101121221120000002200???????????????????????????????????????????????????????????????????????????????????????????????????????????????????????????????????????????????????????????????????????????????????????????????????????????????????????????????????????????????????????????0220011011020020111111110??????1

*Protungulatum* spp.

001???????????????????01010?1?2????10112[0 1]11100010??10011000111022102021002222[1 2]11102111211022102222000011000[0 1]001101111[1 2]1312000100[1 2][1 2]010021200100201001020001?121????????????????????????????????????????????????????????????????????????1????????????????????????????????????00000?011?1?011?10???0??211?0[1 2]?[1 2]001002101011?????????1?00??0201112???0????????????????????000011????????????????????022100001110002110111012000000?0

*Oxyprimus erikseni*

001?????????????????????????1??????????????????????10012000111022102021002222[1 2]1110211121102210222200001?0000001101111[1 2]121200010[0 1]?1010021200100???00??2?????????????????????????????????????????????????????????????????????????????????????????????????????????????????????????????????????????????????????????????????????????????????????????????????????????????????????????????????????????????????????????????????????????

*Vulpavus* spp.

00[1 2]000210000000000000001010?[1 2]?0111?00011230000011??00000000?12022002021002222111102111100?22101111[0 1]10011001100210111122131000010[1 2][1 2]0?0011?00?0000001112??01?1??11[0 1]011001[1 2]010110001111001?0?01[0 2]?????01?0010[0 1]?0??????????0000110?01?01001111??110?0???2120111000?01100?011300000000?0?11010?1?12000102[0 1]1[0 1]0010[0 1]000000100?1[1 2]10102???01?00????????????0??????????00??1?001??000110000[0 1]???10111001???002200010210100201020111000011110

*Miacis* spp.

001000210000000000000?01010?1?0111?0000123000101???00010000012022002021002221011102111100?221001[0 2][0 1]01001100110010011111023100001[0 1]?10100?10000?0????1111???1?1[1 2 3]1?????10012?10?10???????????????20???0???????10?1000000110000?10?010010?111111?101011?21[2 3]0111000?011000111[1 3]00000000?0111010?1?1200011111000???00000010101[1 2]10002???01?00????????????0??????????0010????10?000????0?00??????1?????000????????1?1?0???????1???00111?0

*Gujaratia pakistanensis*

00100021?0000?0400000001110?1?2001?20012001100011??000201001111120020210022221111021111221221022230000100000?02101112222120001002[1 2]010011000000300001[0 1]2000?????1110111010?0011010101100???????1103?03000000??002001001110000100000000011111??1?1010021200?1000101100???1300000000????1?1??1???0?01??????????????02????1?11[0 1]0102?01000????????2??30???????1???010????10011??11?010101??111?12100012210010201201101012101100000000

*Hyopsodus* spp.

001300210100000000000011110?102110?10012001100010??0[0 1]1221100111120020210022221111021120220221012232[0 1]00110[0 1]0000210111222221000100[0 1 2]2010111010000[0 2]01001110?01?1211110111000011110001011?012000[0 1 2]00103?0000000000??00?000??02??011?0?111001111011101010021201?10002011000101100100000?0?1101011?100101002[0 1]?002??00100200101[1 2]101?10???1?00?00201?11???0???111?0?1?02001011100011111010101100?100210000121001021120020101110100001?1?0

*Meniscotherium* spp.

001100210040000400000011110?102001?20012201110011??00022110011212002011102222111112122020122101223210011000000110111222031?0000002110121000000001001120011?121111011[1 2]0100[0 1]011000101100121?0[0 1]02000000?0000001?0001000??000001100?2100011110??101010?[1 2]12011100?2011000101300100000?011101011?10010000110002???0?00210101[1 2]0?111????1?00?0???1??1???0?0111101110020111110010111110101011111100210000120000021000020101111120000?0?0

*Phenacodus* spp.

001000211040000400000001110?1?2010?210?2201110?11??0[0 1 2]0221[1 2]001[0 1]112002011102222211112122022022102223210011000100210111222211000100020100[0 1]1000?00001001120?0?????111011[1 2]0000?01100010110012100220?03?00000[0 1]0000?00000001012??011?0021??011110????1010?212011100?2011000101300100??002?1101011?10010000?1??02??10?002100?12100[0 1]10???1?00?0??????100[0 3]0?01111???10020111?1001011111010101011110121000022?001021?00020101?112?000000?0

*Ptilocercus lowii*

0020003110000021100010101110200000?10011020100?????00022100110012202021002212211100110100?2211112201001000000011021312231200010031010011200000200001020001?131112001011200011110110100100000001110000001111111000000110001110100201110111011101010021201010000011001101111000010?111110231?1210100211110[0 1]111000020010120110010101100100211112??30101111211[0 1]10101101001000110000101110001001100002210110210200201122111101111111

*Plesiadapis* spp.

00[2 3][0 1]1033013000101020-0[1 2]12--?[2 3]?20[0 1]0-2[0 1]012[1 2]011[0 1]0-----0000[0 1]1[1 2]0011122[0 1]02021[0 1]02222[1 2]1110211012202210222320001[0 1]00110021021312210200010032100021200000200011011201-13111201120020111101010010000000000113-01000000110?10???01?02-0010?01?120001111?1112?20011200-11002011100101100002000-211110211-121000031??[0 2]02?211111200201[1 2]11100?10110001???????2--30111111?1??00101?000010001100000?1100[0 1]01001?0?002200110110000201111111111111111

*Notharctus* spp.

0010003200400004000000010100102011?20002[1 2]01100010??0[0 1]02211001112210202110222211110211012212210122320001101?????10212222102000100121[0 1]00[1 2]1200000200001021[0 1]11?13111101100011101100010011010000000113?0000001001?0001?1???0001110?0?[0 1]120001110?11110200?1200?1100?011000001100002010?111110211?12101003111?0[0 1]11?1111200201[1 2]1110010011000?????11?2??30?111110??00010??0?001000110000101100001012100002210100210211101122110111111111

*Adapis* spp.

0013003200400004000000[0 1]11100102010?[1 2]0002201100010??00022110011122[0 1]02021002222[1 2]1110211012002210122320001001?????10212222002?0010012100011000000300101021[0 1]11?13111101[0 1][0 1]0021101100010011010000010113?00000[0 1]0001100011011?0001110?010120001110?1101020011210?11001011000001100002010?111110211?1210100311??0[0 1]11?1111200201[1 2]111001001100010??0???2??30????????????????????1000110?00??11??????02????02210100210[1 2]011011221101111111?1

*Tribosphenomys* spp.

0032114301221?121131?02?2???3?2????0002???????????????0210?010122002021002222211122?101220221022031?001111?????21313122102010001?2100?102100?02??0???10101?131?1???1110????????????????????????????10?????1??????????????????????????????????????????????????????????????????????????????????????????????????????????????????????????????????????????????????????????????????????????????????0002210010110200201011111?0???????

*Paramys* spp.

0032114301221?121131?02?2???[2 3]?2????00022201110????????0211?0101[0 1]33?????1??222211?22?22?2202210222320001111?????2131322210101000[0 1]32100110210000[0 2]??010220101?1311120?1110200011000111110?0100002113?0200000001??3011001?02??010100001000111011102020020201110212001200001300010000?011110001?1010000012000011101002100?11111020?001000?0??????2???0?011110??00010??0?0000[0 1]011?10?00?112111002100002200010110200201011112100000000

*Rhombomylus turpanensis*

0032114301221?121131?02?2???3?2????2[0 1]0?2[0 1]10200?????011220200111233?????0??021201?22?20100?221022032?1?1111?????110131211120000003[1 2]100011210000210000020101?1211120?1110200011000111110?0100002003?01000010111010110010000011010121200011111111202002120[0 1]010212001100002[1 3]000102???211110[1 2]11?12100103120202?2?01002000?1210[0 1]0202111000000201012??201???1??1??0?1?1?0110000111010????110111012100002210010110200211011101200000000

*Gomphos elkema*

00[2 3]21132012110121131102?2???[2 3]?2????210?2100000?????022021200101233?????1??022211?22?201020221022032?0?1111?????1131312210201000032100011210000210010020101?1211120?111010001?01??11110??100?02003?0100001011?010??00??02??01??0??0???01111?1101010??1201?1031100120000210?0002??????1??????????????????????????????????1??0?02101000???????????30??111??????000110?001?011????????10?111?1?10000220001021020111101111100???????

*Mimotona wana*

00[2 3]21132012110121131102?2???[2 3]?2????20002000000?????000021000101233?????[0 1]??022211?22?20100?221022032?0?1011?????11?131221020000003?1??????10???????????01??????11[1 2]0?1110?????1??01?1?????1??????????000000?1????????????????????????????????????????????????????????????????????????????????????????????????????????????????????????????????????????????????????????????????????????????????????????????????????????????????????

*Blarina brevicauda*

00[2 3]3002[2 3]11201[0 1]1110310011[1 2]?1?[2 3]?0????10021200201?????000222?0010011002001102022211110100100?121101022?1111000110111213112332000011?3010020000000000101110101?1311110112011110111101101?0??0?020110100011???21?00?1?????102???12?00?10000111111101010021[2 3]00?1100??10???111300001000?011110031?1200000011010001000002112012101000100100100?101012??00?01111001[0 1]00001001100000110000011100101001100102200011210000201011101?10000100

*Erinaceus europaeus*

00[2 3]0002211000[0 1]10100010101110[2 3]00????[0 1]00[0 1]1000100?????00022000010012002021000222211?02100100?22010223201111000100211213122032?0011[0 1]3[2 3]010121000000300101020101?131112001001111111111??01101200100111100100101111?0101110[0 1]002??010010002000111111101010021200?104020111?1100111001000?011110011?1010100210000011001000111012101020100[0 1]000100201?1110[2 3]0101111101100101101100110110000111102011001100102210110111201201010101100000000

*Solenodon* *paradoxos*

002300210130012000000010110?2?1000?10011020100?????00012000010020002020012213??1120111100?120100011?0010000000201001000030?0000013010021100000301111010101?131111011[0 2]111111111101111001200001111100001???211101011100002???1000000000111110110100?02120111100?0110?1211300000000?011110011?1100000011000111001002112012110000110100110??????2??30101110101110201101010000110101001100101001100002210010211?0020101010211??10110

*Eoryctes melanus*

002?00?[2 3]??????????0?????01??2?1????10011220001?????0001000?0100220020210022[0 1]0101102112000?120?0100??0?100000?02000110203?20000?0[2 3]2????????????????????010??????????1001???0?10?11?110?????0?2210100?0????2???????00?0??2???10?00?00?001111?11??0???2120111100?0110?1[1 2]0111101?010?1?1110????1210100211000[0 1]1100100011101?1000?01?01???????????????0??????????????????????????????????????????????????????????????????????????????

*Potamogale velox*

0023002101200023000010101100202000?20012210000?????11012001110021102022102212001100111100?22000100??0010000000211111020032?0000013010020000000000001010201?131111011210111011111??111?1000032100000001???210?00100001002???1001101101011110110100?02120111100?0110?0211211101000?011100011?110000111101011100100010201210100011010011002011110000101111101110001010110110110000011110010?02100101200?11110201??1?00102?0000?010

*Orycteropus afer*

01[2 3]???54??????????????2?2???[2 3]?2???????0??????0????????????0?10?0??????????????????????????????????????????????????????????????100[1 2 3]010110010000300101020201?1311001?[0 1]201011111010010120?2000401101010000001000001000001000001010000000011100110100?021200?10412010??0201100020000?011101011?1000000011100110101002100?111110001101000102202?12??[1 2 3]01?11110??000201101111001110001010101111102?110122010111100002?101?112????????0

*Rhynchocyon* spp.

00210051?????103100011001000102001?12011220201001??11[0 1]221200222033?????0??021211?02?10100?22101100??0?1??000011103122220?2?000?012011100000000300101020201?131110011001101111010011100120000210100100001010000010000010010010111020000112101101011021200?104020010101011110?1010?111100031?101010011202?011?0???10?201[1 2]?100001111101100211112??20101111101000101111110101110001010100111012111112211011211201221010102010000000

*Procavia capensis*

00121[0 1]4201221?031000102?2???1?2020?22032201200000??121221100102033?????1??021211?12?12300?22101123202310100001011111122032?0100001111120010000301101020211?1211111?1110001111000101100?2000[0 1]0[0 1][0 1]1001[0 1]00000001101011000[0 1]0010110101200010112111101011021211010410010??02011001001?00211110011?10000000120002?2100002000?021010102111100102100112??01101111001100201120100111111001010101111[0 1]12101102201111101201221030102200000000

*Moeritherium trigodon*

00211[0 1]2202000102100010112???2?2????22012201100?????121221110111033?????0??022211?02?02300?2210122321001011?????11214222102001001?1101131010100001001020011?1??1110111?0100011001??????000001[0 1]1100002001010?1?1?001101002??010?01000100111001101000011?11010400000??0001?00???????????01?????????????????????0??02??????102?0???01?0???0?12?0????1?0111100?11020112011011011????????00110001????????????????????????????????????

*Chaetophractus villosus*

01130054?????0????????1111?????????????????????????????????????????????????????????????????????????????????????????????????????000000120010100300101120201?131110111?10100101010100100100103?100200?00000001101011001102??01020012010011111110200?121200?103120111112011000101?01211110121?11000001221002?2001002000?110111002111100100200010100010111101111121110010100011000101110110100210110220000011020020101110010???????

*Bradypus variegatus*

0133??54??????????????112??????????????????????????????????????????????????????????????????????????????????????????????????????003100120010100000001021211?131100102??011[0 1]011000110100020003?000200?00000201100011001102??01020100010011201110200?021[2 3]00?11002010??00011000001?01201110021?11000011121002?2001002000?1200[0 1]0002101110100102?110100101111010111211010000010110000011112010002100003200010100201121010010000011110

*Tamandua* spp.

1??????????????????????????????????????????????????????????????????????????????????????????????????????????????????????????????1?00?0?000?0100000111020201?13111?1?1?01101111010110[0 1]00?20003?10020??001002?0000000000102??0102010201001120?111200?021200?11002010??0100100000000?211110??1?1?0000???????2?2?0?002000?12101100210110010020101[1 2]0100?01111001111211000001000110001011110010002100002200110100200221010110001110110

*Dilambdogale gheerbranti*

002??????????????????????1??2?0???????01000000???????0120100100[1 2]11000001122110001101101[0 1]2022001200??001100010010[0 1]3110111010000[0 1]02301??????0????????????????????????100???????????????????????????????????????????????????????????????????????????????????????????????????????????????????????????????????????????????????????????????????????????????????????????????0?0111?????????????????????????????????????????????0?1?1??

*Widanelfarasia bowni*

002?00?1????????????????01??2?0000?00101000000?????000120100100211000100?221000011011010202210200???0011000100[1 2]0131101110100000???01012?100?0000100100??01?1???????100?????????????????????????????00??????????????????????????????????????????????????????????????????????????????????????????????????????????????????????????????????????????????????????????????????????????????????????????????????????????????????????????

*Todralestes variabilis*

002?????????????????????????20????????01000000?????00022000?100220010000022201001011112[0 2]201200122100001100010011031[1 2]11110[1 2]00000033010?[2 3]?200??000?10?01??01?13??????100???????????????????????????????????????????????????????????????????????????????????????????????????????????????????????????????????????????????????????????????????????????????????????????????000011?????????????????????????????????????????????00101??

*Microgale talazaci*

0021002111100020000010010100200000?10010000000?????00012001?11020200010012203??012??1??000210000000?00110001001013010000300000013?010130000000000001000201?1211110011100110101101101?00100020010000001???211?01111101002?001021001100011111111101112121101100?011001012201100000?011100011?1000001?210111?1001012?12011101000000100010?21001???0010111?101100?010011[0 1]000[0 1]110000011110111?0?10011220011011[0 1]200211100010[1 2]00110010

*Foxomomys fremdi*

002?0??3???????11?2?????111?2?0????00011221101?????000000[0 1]0111022[0 1]02021002222[1 2]111021101121121122220000110001001001131221120001002200?0??200000??????????????31?????????????????????????????????????????????????????????????????????????????????????????????????????????????????????????????????????????????????????????????????????????????????????????????????????????????????????????????????????????????????????????????????

*Dryomomys szalayi*

00210033013000111020?0?0111?2?0000?20112221101?????0000001011112210202100222211110211012212211222200001100000011011312210200010032000021200000200011021101?1311110111110???1??00?0?1??????0?2??????000???01????????????2?0?????????0???1?????????????????1100?01110?1????????010?0111102?1?12?0?0?????????????01?00201??????10??1??0??????????????????????????01???000000????000???0000??01?0?00220011011020020111211110?????11

*Tinimomys graybulliensis*

00310033013000111030?0?0111?[2 3]?0000?2[0 1]012221101?????000[0 1]001011112210202100222211110211012212211222211001100010011011312210200010032000021200000200011021101?1311???11111???????????????????????????000?????1????????????????????????0?????????????????????????????????????????010?0111102?1?12?0?0?????????????01?00201???1????????????????????????????????????0????000000110000001100001001?00002200110110200201112111101111111

*Carpolestes* spp.

00[2 3]?0022013000111020102?111?[2 3]?0010?21112200011????????00???01112210202100222211110211012212211122210001000110021011312210200010132100021200000200011011201?131111011011201011010100110??001?00103?00000010111100??00??02?0010?0??020?0?1?1?11?20200112?0?1100201100110?11?002000?0111102?1?12?0?0?31??????????01200201?01100???0???0????????2???0?111???????0?0????00100011000?0?1100001001?0?002200110110200201111111111111111

*Ignacius* spp.

00321033013000111030?0112???3?2000?00002101100????????0001?011122102021002222111102110120?2211122300001000??00211113122102000000321100212000002??0????1201??3111101111000101101010110000000021103?01000010111100??0???02?00??????120?0?111?1112020021200?1100201110110?110010010?2111102?1?12?010??11???????0?01200201??110010?011?01????1??2??10?111??????0010??0?00100011000?0?0100001001?00002200110110100201111111111111111

*Cynocephalus volans*

0033003112[1 3]000241000101010003?2020?02012200010?????00102121110222102021002222[1 2]111101101220221122000?001000010021031322230200001002110121100000201001111211?13111111110000101111001110012000002113?000000001001000000110000110100021110111011111111021310?11000010??0011100010????211110011?11000010111102-?10?01000101101100021111001??211112??001111112?0[0 1]00211101000001110000101100011001100002200110210200201111111100110011

*Plagioctenodon rosei*

00100021?0[2 3]00?0[1 4]10001001011?1?0010?11111111100010??10022020010012102001000201210102100112112011121100010000[0 1]001111121122120000[0 1]02[1 2]010121200??000000101?2?1?1311110?1[0 1]011???111?1??11???????????????00011?111?0??11?????2?0??????????????????????????????????????????????00?00000?011?1?0?1?1???00???????10100100211101???1?0010??00?????0111???20101111?????0?0????000000110000??11???01001?00002210110210200201122101110110110

*Leptacodon tener*

001???????????????????01????1?001??101?11???00????????1???0?1001210200100?21121?10?100112112011?211000100000001111121122120000[0 1]0?[1 2]01012?200000200001?1???1?1???????????????????????????????????????????????????????????????????????????????????????????????????????????????????????????????????????????????????????????????????????????????????????????????????????????????????????????????????????????????????????????????????

*Plagioctenodon thewisseni*

001?00?1??????0?100?1001011?1?001??11111111100010??100220200100121020210002012101021001121120111211000100001001111121122120000[0 1]02[1 2]01012120000000000101???1?131?1?0?100?????????????????????????????00??????1?0??1??????????????????????????????????????????????????????????01000?011?1?0?1?1????0????????0??0101211101????????????????????11???20???????????0?0????000000110?00??11???0?0???????????????????????????????011011?

*Adunator minutus*

001?00?1???0????100?10??01??1?0????110111?0100010??1002211001001200202100021121?1021001221120?1?11100011000000111112112212000010220????????????????????2?1?????????????????????????????????????????????????????????????????????????????????????????????????????????????????????????????????????????????????????????????????????????????????????????????????????????????????????????????????????????????????????????????????????

*Macrocranion junnei*

001??0?????????????????????????????100?1020000????????1100?01001210202100[0 2]2111101021001221120112111000?1000000111112122212000010?20??????????????????????1?????????????????????????????????????????????????????????????????????????????????????????????????????????????????????????????????????????????????????????????????????????????????????????????????????????????????????????????????????????????????????????????????????

*Macrocranion nitens*

00100021???00?0[1 4]10001001111?1?0010?10011020000010?????1?0?001001200202100[0 2]21221?1021001221220112?210???0000100011?121222120000102201?????0???????????????1????????????111???????????????????????????????????????????????????????????????????????????????????????????????????0000?0?1?1?0?1?1???1????????????0100??1101??????????????????????????0??????????????????????????????????????????????????????????????????????????????

*Microsyops annectens*

002[0 1]003?1100001?1020?010????2?2000-10012201100----????1212?0111121020211022221111121001221221122231000100001002103131223220001013-100??1?0????????????1201-???1100111000??01101001110012000???????000000000001000000?00000?11?0001?0?01111???1?1??011210-1100201100011?111000000?011110011-11100?0?1111011?10100000101101100????1???10??01??2??0???????????????????????????????????????????????????????????????????????????????

*Zanycteris paleocenus*

0021??????????????????01????2??000-???0?????????????????????12112012021002222111102110100-220112130000110??????????????????????????????????????????????????????????1010???????????????????0???10??0100?1??1????????????2?0????????????????????????0??2?11?1?????????[1 2]???1??01000?011?1?011-1[1 2]?0?0????0?0???001?0?11100[1 2]?????0[1 2]?????????21101???????????????????????????????????????????????????????????????????????????????????

*Picrodus calgariensis*

00[1 2]?00?2??????1110?0?0112--?[1 2]?2011-00001000100-----???0010?012122012021102222111102110100-220112130000110001000002131221?10000113-1???????????????????1201-????????10?0????????????????????10??????100?11?1????????????????????????????????????????????????????????????????????????????????????????????????????????????????????????????????????????????????????????????????????????????????????????????????????????????????????

*Draconodus apertus*

00???????????????????????????????????????????????????????????2122012021002220111102110100-2201121300???????????????????????????????????????????????????????????????????????????????????????????????????????????????????????????????????????????????????????????????????????????????????????????????????????????????????????????????????????????????????????????????????????????????????????????????????????????????????????????

*Labidolemur kayi*

00[2 3]-0033013000111030-02-2--?[2 3]?2011?00001020000-----00-00000010012112021002201111100110100-220102021-001000010001031211210100000[0 1]3310001121000000001010??01-1??1120?12100110100101111?0??010??10???0000?1111?????11????02-001??????10?0?1???????00-0??211111002011001011111000000?011111011-121010001101001?0010020010111010?0100100?0??211012???0??????????????????????????????????????????????????????????????????????????????

*Plesiolestes nacimienti*

002???????????????????1111??2?1000-00002121100--???000100?0?10122102021002222111102110122122112222000011000100110213122112000100?211???1???????????????????????????10100??????10?1?1??????0??111??0000?????????????????20??????????????????????????????????????????????????????????????????????????????????????????????????????????????????????????????????????????????????????????????????????????????????????????????????????

***Mixodectes pungens***

**002-0032?10000111020102-2--?2?0001-00011001100-----00000010111022102021102221110110110122122111221100011000100210313122222000000[2 3][2 3]110??1200000200000001201-?31?1???11?0???????????????????0???1???0000???00????????????000?????????0?0?1??????????????????00?2?11001?????????00??????1?01???1??????????????????????????????0?????????????????????1???110????0101???0010001100000???00001001?00002200110110200201111111101111111**

Character-state revisions to Crowell et al. (2024) matrix

Character 344

- *Cynocephalus* ? 🡪 2
- *Notharctus* ? 🡪 0
- *Ptilocercus* ? 🡪 2

Character 358

- *Adapis* ? 🡪 1
- *Plesiadapis* 0 🡪 1
- *Ptilocercus* 0 🡪 1

Character 360

- *Adapis* ? 🡪 0

Character 361

- *Adapis* ? 🡪 0

Character 362

- *Adapis* ? 🡪 1

Character 368

- *Ptilocercus* 0 🡪 1

Character 372

- *Ptilocercus* 0 🡪 1

Character 378

- *Adapis* ? 🡪 0
- *Notharctus* 0 🡪 1

Character 390

- *Protungulatum* ? 🡪 0

Character 395

- *Adapis* 0 🡪 1&2
- *Notharctus* 0 🡪 2

Character 396

- *Adapis* 1 🡪 0

Character 398

- *Ptilocercus* 1 🡪 2

Character 399

- *Plagioctenodon rosei* 2 🡪 0

Character 403

- *Ptilocercus* 1 🡪 2

Character 406

- *Adapis* 1 🡪 0
- *Notharctus* 2 🡪 0

Character 407

- *Adapis* 0 🡪 1
- *Carpolestes* 0 🡪 1
- *Cynocephalus* 0 🡪 1
- *Dryomomys szalayi* 0 🡪 1
- *Ignacius* 0 🡪 1
- *Notharctus* 0 🡪 1
- *Plagioctenodon rosei* 0 🡪 1
- *Plesiadapis* 0 🡪 1
- *Ptilocercus* 0 🡪 1
- *Purgatorius* 0 🡪 1
- *Tinimomys graybulliensis* 0 🡪 1

Character 412

- *Cynocephalus* 1 🡪 0

Force commands in modified Crowell et al. (2024) constrained analysis

force +

[68 69 70 71 79(4 5 6 7 8 9 10 11 12 13 14 15 16 17 18 19 20 21 22 23 24 25 26 27 28 29 30 31 32 33 34 35 36 37 38 39 40 41 42 43 44 45 46 47 48 50 52 53 54 56 59 61 62 63 67 72 76 77 78 80 81 82 83 84 86 87 88 89 90 91 92 93 94 95 96 97 98)]

[73 74 75(4 5 6 7 8 9 10 11 12 13 14 15 16 17 18 19 20 21 22 23 24 25 26 27 28 29 30 31 32 33 34 35 36 37 38 39 40 41 42 43 44 45 46 47 48 50 52 53 54 56 59 61 62 63 67 72 76 77 78 80 81 82 83 84 86 87 88 89 90 91 92 93 94 95 96 97 98)]

[64 65 66 49 51 55 57 58 85 60 (4 5 6 7 8 9 10 11 12 13 14 15 16 17 18 19 20 21 22 23 24 25 26 27 28 29 30 31 32 33 34 35 36 37 38 39 40 41 42 43 44 45 46 47 48 50 52 53 54 56 59 61 62 63 67 72 76 77 78 80 81 82 83 84 86 87 88 89 90 91 92 93 94 95 96 97 98)]

[64 65 66 49 51(4 5 6 7 8 9 10 11 12 13 14 15 16 17 18 19 20 21 22 23 24 25 26 27 28 29 30 31 32 33 34 35 36 37 38 39 40 41 42 43 44 45 46 47 48 50 52 53 54 56 59 61 62 63 67 72 76 77 78 80 81 82 83 84 86 87 88 89 90 91 92 93 94 95 96 97 98)]

[55 57 58 85 60(4 5 6 7 8 9 10 11 12 13 14 15 16 17 18 19 20 21 22 23 24 25 26 27 28 29 30 31 32 33 34 35 36 37 38 39 40 41 42 43 44 45 46 47 48 50 52 53 54 56 59 61 62 63 67 72 76 77 78 80 81 82 83 84 86 87 88 89 90 91 92 93 94 95 96 97 98)]

;

Cladistic methodology and results of modified Crowell et al. (2024) matrix

Cladistic analyses were conducted in TNT (v.1.5) [14] on a matrix of 415 characters and 99 taxa derived from [7]. One new taxon, the mixodectid *Mixodectes pungens*, was added, and character codings were revised based in part on new observations from micro-CT data (see Character-state revisions section above). Search methodology generally followed that of [7] for unconstrained and constrained analyses. For the constrained analysis, the monophyly of Afrotheria, Xenarthra, Boreoeutheria, Laurasiatheria, and Euarchontoglires were enforced (see Force commands section above), which are well supported clades in virtually all recent phylogenetic analyses of placental mammals based on molecular data [e.g., 15]. Although equal-weights parsimony analyses were performed here, other phylogenetic methods (e.g., Bayesian inference [16] and implied weighting for parsimony [17]) can provide future tests of the relationships among mixodectids and other eutherian mammals.

A New Technology search was performed to initially search tree space in the unconstrained analysis, which recovered 917 most parsimonious trees (MPTs) with lengths of 2,925. These resulting trees were then used in a heuristic search that resulted in 940 MPTs with lengths of 2,925. Following the identification and removal of *Khamerungulatum* using the pruntaxa function, the number of MPTs was reduced to 200 with lengths of 2,925. A strict consensus of these results (Fig. S2) is well-resolved but does not support commonly recovered placental mammal interrelationships. For example, Laurasiatheria is polyphyletic and a monophyletic Euarchonta is the sister group to a clade containing Glires, picrodontids, the apatemyid *Labidolemur kayi*, lipotyphlans, afrosoricids, *Eoryctes*, and the Cretaceous eutherian *Lainodon*. Within Euarchonta, the treeshrew *Ptilocercus* is sister to Primatomorpha (Primates + Dermoptera) with several plesiadapiforms recovered as stem primatomorphans, including *Purgatorius*, micromomyids (*Foxomomys, Tinimomys,* and *Dryomomys*), and the palaechthonid *Plesiolestes*. Other plesiadapiforms, such as the paromomyid *Ignacius*, carpolestid *Carpolestes*, and plesiadapid *Plesiadapis* are recovered as stem primates. *Mixodectes pungens* is recovered as the sister taxon to the microsyopid *Microsyops annectens*, and both taxa are supported as the sister group to the extant colugo *Cynocephalus*.

A New Technology search was performed to initially search tree space in the constrained analysis, which recovered 8,422 MPTs with lengths of 2,950. These resulting trees were then used in a heuristic search that resulted in 10,000 MPTs with lengths of 2,950. *Acristatherium*, *Montanalestes*, *Lainodon*, and *Khamerungulatum* were identified as wildcard taxa and were subsequently removed using the pruntaxa function, which ultimately decreased the number of MPTs to 2,976 with lengths of 2,914. A strict consensus of these results is well-resolved, and, like the results of the unconstrained analysis, Euarchonta and Primatomorpha are recovered as monophyletic (Fig. S3). Unlike the results of the unconstrained analysis, *Mixodectes pungens* is recovered as a stem primatomorphan. All non-microsyopid plesiadapiforms are supported as stem primates, and the microsyopid *Microsyops annectens* is supported as the sister to the extant colugo *Cynocephalus*.

Synapomorphies resulting from the unconstrained analysis modified from Crowell et al. (2024)

Euarchonta

44: 0 🡪 1 Ultimate upper premolar postcingulum present, lower than protocone

55: 1 🡪 2 Ultimate lower premolar paraconid distinctive and high

60: 0 🡪 1 Ultimate lower premolar anterolingual cingulid present

192: 0 🡪 1 Posterior nasal spine prominent

280: 0 🡪 1 Intratympanic course of facial nerve open anteriorly, canal posteriorly

294: 0 🡪 1 Fossa incudis separated from epitympanic recess

300: 0 🡪 1 Stapedius fossa small and shallow

306: 1 🡪 0 Caudal tympanic process of petrosal notched absent

312: 1 🡪 0 Jugular foramen subequal relative to fenestra cochleae

313: 0 🡪 1 Jugular foramen separated from opening for interior petrosal sinus

317: 0 🡪 1 Ectotympanic aphaneric or hidden by auditory bulla

318: 1 🡪 0 Ectotympanic ringlike

322: 0 🡪 1 Entotympanic present

374: 1 🡪 0 Greater trochanter lower than femoral head

401: 0 🡪 1 Calcaneal sustentacular facet expanded onto body

409: 0 🡪 1 Lateral keel of humerus present

410: 0 🡪 1 Zona conoidea of humerus present and narrow

415: 0 🡪 1 Plantar pit on calcaneal cuboid facet present

Primatomorpha

42: 2 🡪 0 Ultimate upper premolar para- and metastylar lobes absent or insignificant

98: 2 🡪 3 Postcingulum present, extending to labial margin

104: 1 🡪 0 Ultimate upper molar width subequal to penultimate molar

111: 1 🡪 2 Trigonid anteroposteriorly compressed, paracristid-protocristid angle 35 degrees or less

*Cynocephalus +* (*Mixodectes* + *Microsyops*)

9: 0 🡪 1 Anteriormost upper incisor alveoli separated by broad gap

82: 0 🡪 1 Centrocrista v-shaped

114: 1 🡪 3 Anterio and labial (mesiobuccal) cingular cuspule absent

120: 1 🡪 3 Hypoconulid in close approximation to entoconid

166: 1 🡪 0 Infraorbital canal long (more than one molar length)

177: 1 🡪 0 Large, triangular and anteriorly pointed facial process of lacrimal

184: 0 🡪 2 Premaxillary-maxillary suture on palate is wedge shaped, pointing posteriorly

201: 1 🡪 0 Jugal-lacrimal contact present

204: 1 🡪 0 Palatine reaches infraorbital canal

205: 1 🡪 0 Lacrimal contributes to maxillary foramen

216: 2 🡪 0 Postorbital process present and prominent

240: 0 🡪 1 Midline rod-shaped eminence on basisphenoid present

247: 0 🡪 1 Foramen ovale on ventral surface of skull

262: 0 🡪 1 Posttympanic crest of squamosal present

280: 2 🡪 0 Intratympanic course of facial nerve open in sulcus

285: 2 🡪 1 Length of bony shelf lateral to promontorium confined posterolaterally

305: 2 🡪 0 Paroccipital process vertical

336: 3 🡪 0 Mastoid foramen absent

*Mixodectes* + *Microsyops*

11: 3 🡪 0 Anteriormost upper incisor shape, conical

72: 0 🡪 1 Mesostyle present

99: 0 🡪 1 Hypocone on postcingulum present, lower than protocone

121: 0 🡪 2 Hypoconulid of ultimate molar posteriorly procumbent

203: 1 🡪 0 Roots of molars exposed in orbit floor absent

Stem Primates + Euprimates

60: 1 🡪 0 Ultimate lower premolar anterolingual cingulid absent

95: 2 🡪 1 Degree of labial shift of protocone, moderate labial shift

178: 1 🡪 0 Lacrimal tubercle present

227: 1 🡪 2 Frontoparietal suture with anterior process of parietal on midline

239: 1 🡪 2 Entopterygoid process approaches ear region

241: 1 🡪 2 Ectopterygoid process approaches ear region

408: 0 🡪 1 Deep groove for the tendon of flexor fibularis on calcaneum present

Synapomorphies resulting from the constrained analysis modified from Crowell et al. (2024)

Euarchonta

44: 0 🡪 1 Ultimate upper premolar postcingulum present, lower than protocone

157: 2 🡪 3 Mandibular foramen recessed dorsally from ventral margin, at or above alveolar plane

192: 0 🡪 1 Posterior nasal spine prominent

206: 0 🡪 1 Groove connects maxillary and sphenopalatine foramina

216: 2 🡪 0 Postorbital process present, prominent

294: 0 🡪 1 Fossa incudis separated from epitympanic recess

312: 1 🡪 0 Jugular foramen subequal relative to fenestra cochleae

313: 0 🡪 1 Jugular foramen separated from opening for interior petrosal sinus

318: 1 🡪 0 Ectotympanic ringlike in shape

322: 0 🡪 1 Entotympanic present

358: 0 🡪 1 Sigmoidal shelf for supinator ridge extending proximally from ectepicondyle

374: 1 🡪 0 Greater trochanter lower than femoral head

375: 1 🡪 0 Lesser trochanter large in size

401: 0 🡪 1 Calcaneal sustentacular facet expanded onto body

409: 0 🡪 1 Lateral keel of humerus present

410: 0 🡪 1 Zona conoidea of humerus present and narrow

412: 0 🡪 1 Trochlea of humerus wide relative to proximodistal length

415: 0 🡪 1 Plantar pit on calcaneal cuboid facet present

*Mixodectes* + Primatomorpha

19: 0 🡪 2 Anteriormost lower incisor root extending posteriorly below penultimate or ultimate premolar

43: 0 🡪 1 Ultimate upper premolar precingulum present

58: 0 🡪 1 Two ultimate lower premolar talonid cusps

62: 0 🡪 1 Molar series have posterior size increase

88: 0 🡪 2 Paraconule prominent, midway or closer to paracone

89: 0 🡪 2 Metaconule prominent, midway or closer to metacone

111: 1 🡪 2 Trigonid anterioposteriorly compressed, paracristid-protocristid angle 35 degrees or less

151: 0 🡪 1 Mandibular symphysis deep

202: 1 🡪 0 Zygomatic arch stout

Primatomorpha

40: 1 🡪 2 Ultimate upper premolar protocone approaches paracone in height

41: 0 🡪 2 Ultimate upper premolar metacone large

63: 0 🡪 1 Molar cusp form is inflated, robust

251: 0 🡪 1 Glenoid fossa partly on braincase

*Cynocephalus* + *Microsyops*

58: 1 🡪 2 3 ultimate lower premolar talonid cusps

95: 1 🡪 2 Substantial labial shift of protocone

166: 1 🡪 0 Infraorbital canal long (more than one molar length)

177: 1 🡪 0 Large, triangular and anteriorly pointed facial process of lacrimal

184: 0 🡪 2 Premaxillary-maxillary suture on palate is wedge shaped, pointing posteriorly

201: 1 🡪 0 Jugal-lacrimal contact present

204: 1 🡪 0 Palatine reaches infraorbital canal

205: 1 🡪 0 Lacrimal contributes to maxillary foramen

240: 0 🡪 1 Midline rod-shaped eminence on basisphenoid present

247: 0 🡪 1 Foramen ovale on ventral surface of skull

260: 1 🡪 0 Suprameatal foramen absent

262: 0 🡪 1 Posttympanic crest of squamosal present

305: 2 🡪 0 Paroccipital process vertical

336: 3 🡪 0 Mastoid foramen absent

Stem Primates + Euprimates

114: 3 🡪 1 Anterior and labial cingular cuspule present, with a distinct cingular shelf posteroventrally directed from it

120: 3 🡪 1 Hypoconulid in postermedial position near the midpoint of transverse talonid width

147: 0 🡪 1 Root of angular process anterior relative to condylar process

178: 1 🡪 0 Lacrimal tubercle present

216: 0 🡪 2 Postorbital process absent

227: 1 🡪 2 Frontoparietal suture with anterior process of parietal on midline

239: 1 🡪 2 Entopterygoid process approaches ear region

241: 1 🡪 2 Ectopterygoid process approaches ear region

280: 0 🡪 2 Intratympanic course of facial nerve in canal

308: 1 🡪 2 “Tympanic process” present and high

Character-taxon matrix modified from that of Chester et al. (2017)

Modified character-taxon matrix of [18] (originally derived from [19] and subsequently modified by [12,18]). This modified character-taxon matrix is provided (with the additional taxon, *Mixodectes pungens*, in bold) in TNT format below and is freely available in the Morphobank.org repository, <http://morphobank.org/permalink/?P5501>. The character and character state definitions are provided in [19] and are also freely available on Morphobank.

*Ukhaatherium nessovi*

10100010000000000000??00?100110010000000020020000?1001?000101000?000000110?0210?11100?101??021?00110?0000?00000000000010002002100101020001000001100000000102?00011100011000020010000000010101000000002010001200200110000001000000000200000000000

*Erinaceus europaeus*

20010110200021001100000100101000100102?002001211010011?00010000000201121000110021100101100012100011101000?0010001100001110102001010002100011111110101101100011110011300011001011100000000010110012?11200121131111010100011???2000020002000200000

*Echinosorex gymnure*

210101112000?10012000011002110011001??01020002110000110000100000001001210000100201001011000110000110?1000?0010001100001010100210010102100111100110101101110011110011300011011010011000000010000001001200001032110010100011???2000000002000200000

*Hemiechinus auratus* 210110102000210000????0110100000100112?????????????????????0???01020102100012012[0 1]100101110012100011101000?00100011000011102002000101021001111101101011011100111100112?0011011011101000000010110012?112000010321100102000110000000020002000300000

*Solenodon paradoxus*

10110111011000001200000110100010101111011201010100000000001000001010012010000100010100[0 1]00001020?011100000?001001[0 1 2]1000010100?10110000020102010001101011011002?000?11?2?0101001001?000?000201110000002?23012113012001100012????2000100201000000000

*Suncus murinus*

201111000110?100??11000120001010101102?102011211000011000010100?00100020100001001100010000010200011100000?001100?1110021011020010000020001011001101000010110000200112?0001010011100130000110210112?2?0310211301000100000111020000000101000200000

*Sorex* sp.

2011100001000000?2????111010101010010?00020102?[0 1]0000110000101000002000001000010010000?0000000200011100000?00110001110010011020010000021001111001101010010110010200110000010100111001300?0111210112?2?0311211301000100000111020000000101100200000

*Vulpavus profectus*

?010000100000?0?0010??21??100?000010000020000001020000?1?00011?00??0011100?11000??0?01000?1112000?00?0002?0010?0?00000100020001101000200100110011010110000010000010120000101?011000000001000100000001200011121100010000000???2000010002000000000

*Uintacyon rudis*

????????????????????????????????????????????????????????????????????0??1?????????????????????????????????????0?0?????0?0002010110100020012011001101010000000000001101010000120????000000????100000000200011110120010000020???2000000012000000000

*Genetta* sp.

20100011000010000110001102101001001000011200011102000000000001000030122100101[0 1]0100010[0 1]01011020000100?1002?02001010000010002002100101020012011001101010001000?001010?2?00000021?1??0000001000100100000200121131120010000000???2011???002000000000

*Pararyctes pattersoni*

?????????????????????????1?????????110??????????????????????????????02?000??1?020?0?0??11??011?0011101?00?0210?10??????0111012000100020002011001100000000100000010102?1101000001000000003???0100110112000000200200110000200000000101111000000000

*Paleoryctes* sp.

???????10??0????????????????????????????????????????????????????????0??0??????0?????0??01???12?????10???????????1???????01200200110002000001110?100010000102?000111?00110000200100000?0020000000110002100001200200010000200000000000211000000000

*Ottoryctes winkleri*

???????????????????????????????????????????????????0????????????????021[0 1]00??2?0?010100011?001101?111011110021001100??010112102101100020001011000100000000000000111101011000020010000000020000000110002100001200200110000200000000100211000000000

*Gypsonictops* sp.

????????????????????????????????????????????????????????????????????????????????????????????????????????????????????????????0110110102000201100013100001?100000?0??001100000200100000?????????0?000012101000[0 1]000101110001???00000000201010000000

Leptictidae

?01100010000100000?1??210?211011100110011000010102000000001?10000?101221001100020101000101001110000101100?02200110?0?0110020021010210200020100000300000001000010001001100000001100000000100010200000120[0 1]10000000001100000?0000000000300000000000

*Rhombomylus turpanensis*

20000111000?100?00?0?0?1??201001100010?111000100110?00??00??1?0?00??12211100200201000??01?0000?1021100122?0010?0120?1101210?0010[0 1]10102110101101[0 1]101000100102?12001112?011101100001102?113???212212?002001000000000022000011101000101101111000111

*Ptilocercus lowii*

10100001101100010010??10110?000000000010000020010201000101001011021111221011100101000100010010011010011111021100110000211120001000010210010?11010010111[0 1]1101011200112?00010100110000000010100000110112301[0 2]01301000100000011020000000001010200000

*Tupaia glis*

100100010000000000100021022100110000001000002201020100000100100002?002221011200200000000110011111010?111110210001100002101200210000[0 1]020001011101000011101112?00201102?00010000110001300010100000110012301[0 2]01301000100000111020000000001010300000

*Cynocephalus volans*

1010001000100111001010001100000000000000010020000210011001001021103112111011200211000000010110?10200?1022?01011010000020110?02111000021002011110031000000112?1020100000100012000000030001?10001010?012101100001100100100101120000010002000301000

*Purgatorius* sp.

???????????????????????????????????????001002001021000???????????????????????????????????????????????????????????1120???????????????02000101100000000010010000000000000[0 1]00[0 1]0000100000?0?0?1?1000000012000000101000[0 1]10000[0 1]0000000010110[0 1]1100000?0

Microsyopidae

???????????10?????????????????????????????????????1?????????????????11111010210?1000000101111[0 1]11??0101000?0?1000111100211120100100000100?1111001000001110100011001100100011000110010000003102101110012011000111001110?00100110000001301110000000

Micromomyidae

001000010010000101001110110002?01000000001002001021000000?00102???101??100?02???????????1???21?1101???002?1??0?011120021112002110000020001011000001000000100000000000100000000010010000001102101100012230000[1 3][0 1]10010100001[0 1]0110100101300110[0 1]00000

Paromomyidae

?1100001101000010000111112100100000000000000200102[0 1]00000000?102?01?0121100?0211211??0??111112101101101102?12200011130011012?001?000002100[0 2]011[0 1]0100100110010001000001110000210010001000000[1 2]10110[0 1]100002010000111101111000110000010112102212201000

Carpolestidae

?01000011011000?0000210111100110?0000000010020010210001110011020???0122100002112100?0??01?0021?0??111?100?1??0?0?1130020001001001011021002111010111101100100011001000101012100000010000002111000110202201201201010100110110000000112302110000000

Plesiadapidae

?0100001001000000000001111000100000000000000200102[0 1]00001000?001000101221010121?210??0??01?0021001?1111102?1?00?011130011012101101011011100011[0 1]1001100110010[0 1]111001010101002100000010200002111101110012320201311[0 1]1010[0 1]110110000000112102111000100

*Altanius orlovi*

????????????????????????????????????????????????????????????????????????????????????????????????????????????????????????002?0210000102000?011001000111?001000111010000010111201000100??01?0?1100000012001000101010100110110000000112102010200000

Omomyidae

?01100011?11000???????????1112111000000001002200021101??1??1????????2?220?0121021?000?011??021101?1111111?10?0?0?[0 1][0 1]100[1 2]1001001110001021000011[0 1][0 1]1000011100100011001000[0 1]010[0 1]1120[0 1]0001000001[0 1]0011000100120010001110101001[0 1]010000000010[1 2]302[0 1]10200000

Adapidae

?01000011011000000002121122?0201000000000000200102110111100110000?10212200011102100000001100211010111111111010?0100000[1 2]100200010010102100101101100001110010[0 1]0110010100000[0 1]11201000100000100011000000120[0 1]1[0 1][0 1][0 1]311010100110100001000101102010[0 2]00[0 1]00

*Torrejonia wilsoni*

?01???011?11000??0????1?1???110??0?000000000?00?021000????????2??????????????????????????????????????????????0?011110????11?1????????210000110100[2 3]10000001000111010101000021000000100000021011001100121[0 1][0 1]1001110101110001000000101121021112011?0

*Plesiolestes nacimienti*

????????????????????????????????????????????????????????????????????????0??02??????????????????????????????????0????????01101[0 2][0 1]1100[0 1]0210000110000[0 2]1010100100001[0 1]000[0 1]0100002120[0 1]000100??00?1?00001100120?[0 1]000101000111000100100010112202111[0 1]00000

***Mixodectes pungens***

**?010000110110000?0????111?101100?00000000000300102100000??0??02????0???????02??????????????1???????????0???????01110002121??02110000021001111111001011100110001101100000010100?1001130000?1?012211?01213011020100011?000100110000111102100000000**

Character-state revisions to Chester et al. (2017) matrix

Character 71

- Microsyopidae 0 🡪 1
- Paromomyidae 0 🡪 1

Character 77

- *Ptilocercus* 2 🡪 1
- Microsyopidae 0 🡪 2

Character 78

- *Ptilocercus* 1 🡪 0

Character 79

- Micromomyidae 1 🡪 ?

Character 80

- Microsyopidae 0 🡪 ?

Character 89

- *Ptilocercus* 1 🡪 0

Character 92

- *Cynocephalus* 0 🡪 1

Character 93

- *Cynocephalus* 0 🡪 -

Character 94

- *Cynocephalus* 2 🡪 0

Character 95

- *Ptilocercus* 1 🡪 0

Character 96

- *Ptilocercus* 0 🡪 1
- *Tupaia* 0 🡪 1

Character 100

- *Ptilocercus* 1 🡪 0

Character 104

- Paromomyidae 1 🡪 0

Character 107

- *Ptilocercus* 1 🡪 0

Character 125

- *Purgatorius* 0 🡪 ?
- *Plesiolestes nacimienti* 0 🡪 1

Character 126-133

- *Purgatorius* rescored as ?

Character 159

- *Plesiolestes nacimienti* 0 🡪 1

Cladistic methodology and results of modified Chester et al. (2017) matrix

Cladistic analysis was conducted in TNT (v.1.5) [14] on a matrix of 240 characters and 31 taxa derived from [18]. One new taxon, the mixodectid *Mixodectes pungens*, was added, and character codings were revised based in part on new observations from micro-CT data (see Character-state revisions section above). The search methodology followed that of [18] using an unconstrained equal-weights parsimony analysis.

A New Technology search was performed to initially search tree space in the unconstrained analysis, which recovered two MPTs with lengths of 1,021. These resulting trees were then used in a heuristic search that did not recover any additional MPTs. A strict consensus of these results (Fig S4) is well-resolved and recovered a monophyletic Laurasiatheria, Euarchontoglires, Euarchonta, and Primatomorpha. Within Primatomorpha, *Mixodectes pungens* is recovered as a stem primate sister to a clade consisting of microsyopids, paromomyoids (Paromomyidae + Palaechthonidae), plesiadapoids (Plesiadapidae + Carpolestidae), and crown primates, with a *Purgatorius* + Micromomyidae clade supported as the most basal primates.

Synapomorphies resulting from the unconstrained analysis modified from Chester et al. (2017)

Euarchonta

29: 1 🡪 0 Femur lesser trochanter medially projecting

33: 1 🡪 0 Tibia no longer than femur

40: 1 🡪 0 Trochlea of astragalus is shallowly grooved

45: 0 🡪 1 Astragalus sustentacular and navicular facet contact on lateral side

130: 1 🡪 0 P3 metastyle absent

145: 1 🡪 0 P4 metastyle absent

197: 0 🡪 1 p/3 paraconid absent

221: 0 🡪 2 m/1 hypoconulid and entoconid twinned (hypoconulid appressed to the entoconid in distolingual corner of tooth)

Primatomorpha

21: 0 🡪 1 Phalanges digit elongation [(Intermediate phalanx length + proximal phalanx length)/humerus length] of digit III or IV, 35-50%

51: 0 🡪 1 Calcaneum plantar pit on cuboid facet present

63: 0 🡪 2 Phalanges flexor sheath attachments on proximal phalanges of the manus or pes substantially ventrally projecting

71: 2 🡪 1 Frontonasal contact, semi-expanded – nasals flare posteriad but do not touch lacrimal

81: 0 🡪 1 Alisphenoid canal for ramus infraorbitalis absent

92: 0 🡪 1 Occipital nuchal crest distinct and large

128: 0 🡪 1 P3 parastyle absent

132: 1 🡪 0 P3 protocone absent

165: 2 🡪 0 Both M1 conules present

231: 1 🡪 2 Trigonids become more mesiodistally compressed from m1 to m3

Stem Primates + Euprimates

22: 0 🡪 1 Digit III Intermediate phalanx greater than or equal to 80% of metacarpal length

94: 0 🡪 1 Squamosal entoglenoid process present but small (smaller than postglenoid)

115: 0 🡪 1 I1 tip strongly recurved

156: 2 🡪 0 M1 precingulum is present and doesn’t connect to postcingulum

185: 1 🡪 0 Lower i1 size much larger than other incisors (or premolars if i2 and i3 are lost)

212: 0 🡪 1 Lower m1 protoconid subequal in height to metaconid

219: 1 🡪 0 Entoconid notch absent on m1

221: 2 🡪 1 Hypoconulid of m1 paired with entoconid (lingual of the central axis of the tooth but not directly appressed to the entoconid)

226: 0 🡪 1 Lower m3 greater in length than m2

228: 0 🡪 1 Hypoconulid on m3 larger than on m1 and m2, but not developed into a lobe

229: 0 🡪 1 Lower molars get progressively larger from m1-m3

232: 0 🡪 1 Lower molar trigonid mesial inflection weak

*Mixodectes* + (Microsyopidae + Paromomyoidea + Plesiadapoidea + Euprimates)

9: 0 🡪 1 Humerus supinator crest projects prominently posterolaterally

12: 0 🡪 1 Medial and lateral keels present in humerus, trochlea and capitulum well-separated

27: 0 🡪 1 Greater trochanter even with femoral head

42: 1 🡪 0 Astragalus medial border less than 90% height of lateral border

150: 0 🡪 1 P4 postprotocrista absent

159: 0 🡪 1 M1 preparacrista angled straight

194: 0 🡪 1 1 alveolus for p2

**Figure S1.**

**Figure S1. (A)** Geologic map of the San Juan Basin, New Mexico, showing Upper Cretaceous through lower Eocene strata (figure modified from [20] fig. 1) and the location of fossil locality NMMNH L-6898 at the West Flank of Torreon Wash. **(B)** Geochronologic chart showing correlations between North American Land Mammal Ages (NALMA; following [3]), epochs, stages, geomagnetic polarity time scale (GPTS; following [21]), lithostratigraphy of the Nacimiento Formation at the West Flank of Torreon Wash, San Juan Basin, New Mexico, and mammal biozonation of the Nacimiento Formation (following [1,4,22]). **(C)** Stratigraphic section and Nacimiento Formation as measured at the West Flank of Torreon Wash with the local magnetic polarity (Pmag) as correlated to the geomagnetic polarity chronology showing the position of NMMNH locality L-6898 (following [4]).

**Figure S2.**

**Figure S2.** Hypothesis of evolutionary relationships of *Mixodectes pungens* and other eutherian mammals. Resulting unconstrained strict consensus cladogram based on modified morphological dataset of [7].

**Figure S3.**

**Figure S3.** Hypothesis of evolutionary relationships of *Mixodectes pungens* and other eutherian mammals. Resulting constrained strict consensus cladogram based on modified morphological dataset of [7].

**Figure S4.**

**Figure S4.** Hypothesis of evolutionary relationships of *Mixodectes pungens* and other eutherian mammals. Resulting unconstrained strict consensus cladogram based on modified morphological dataset of [18].

**References**

[1] Flynn, A. G. et al. Early Paleocene magnetostratigraphy and revised biostratigraphy of the Ojo Alamo Sandstone and lower Nacimiento Formation, San Juan Basin, New Mexico, USA. *Geol. Soc. Am. Bull.* **132**, 2154-2174. (doi:10.1130/B35481.1) (2020).

[2] Cather, S. M., Heizler, M. T. & Williamson, T. E. Laramide fluvial evolution of the San Juan Basin, New Mexico and Colorado: Paleocurrent and detrital-sanidine age constraints from the Paleocene Nacimiento and Animas formations. *Geosphere* **15**, 1641-1664. (doi:10.1130/GES02072.1) (2019).

[3] Lofgren, D. L., Lillegraven, J. A., Clemens, W. A., Gingerich, P. D. & Williamson, T. E. Paleocene biochronology: the Puercan through Clarkforkian land mammal ages in *Late Cretaceous and Cenozoic mammals of North America: biostratigraphy and geochronology* (ed. Woodburne, M. O.) 43-105 (Columbia University Press, 2004).

[4] Leslie, C. et al. High-resolution magnetostratigraphy of the Upper Nacimiento Formation, San Juan Basin, New Mexico, USA: Implications for basin evolution and mammalian turnover. *Am. J. Sci.* **318**, 300-334. (doi:10.2475/03.2018.02) (2018).

[5] Leslie, C. E. High-resolution age constraints and fluvial sedimentology of Late Cretaceous to early Paleocene terrestrial deposits of the southwestern USA. Ph.D. dissertation, Baylor University, Waco, TX. (2018).

[6] Gygi, D. J. Early Paleocene plant community and paleoclimate reconstruction of the Nacimiento Formation from the San Juan Basin, New Mexico. Master’s thesis, Baylor University, Waco, TX. (2022).

[7] Crowell, J. W., Wible, J. R. & Chester S. G. B. Basicranial evidence suggests picrodontid mammals are not stem primates. *Biol. Lett.* **20**, 20230035; 10.1098/rsbl.2023.0335 (2024).

[8] Wible, J. R., Rougier, G. W., Novacek, M. J. & Asher, R. J. Cretaceous eutherians and Laurasian origin for placental mammals near the K/T boundary. *Nature* **447**, 1003-1006. (doi.org/10.1038/nature05854) (2007).

[9] Wible, J. R., Rougier, G. W., Novacek, M. J. & Asher, R. J. The eutherian mammal *Maelestes gobiensis* from the Late Cretaceous of Mongolia and the phylogeny of Cretaceous Eutheria. *Bull. Am. Mus. Nat. Hist.* **327**, 1-123. (doi:10.1206/623.1) (2009).

[10] Hu, Y., Meng, J., Li, C. & Wang, Y. New basal eutherian mammal from the Early Cretaceous Jehol biota, Liaoning, China. *Proc*. *Biol*. *Sci*. **277**, 229-236. (doi.org/10.1098/rspb.2009.0203) (2010).

[11] Goswami A. et al. A radiation of arboreal basal eutherian mammals beginning in the Late Cretaceous of India. *Proc*. *Natl Acad*. *Sci*. *USA* **108**, 16333-16338. (doi:10.1073/pnas.1108723108) (2011).

[12] Chester, S. G. B., Bloch, J. I., Boyer, D. M. & Clemens, W. A. Oldest known euarchontan postcrania and affinities of Paleocene *Purgatorius* to Primates. *Proc*. *Natl Acad*. *Sci*. *USA* **112**, 1487-1492. (doi:10.1073/pnas.1421707112) (2015).

[13] Manz, C. L., Chester, S. G. B., Bloch, J. I., Silcox, M. T. & Sargis E. J. New partial skeletons of Palaeocene Nyctitheriidae and evaluation of proposed euarchontan affinities. *Biol*. *Lett*. **11**, 20140911. (doi:10.1098/rsbl.2014.0911) (2015).

[14] Goloboff, P. A. & Catalano, S. A. TNT version 1.5, including a full implementation of phylogenetic morphometrics. *Cladistics* **32**, 221-238. (doi:10.1111/cla.12160) (2016).

[15] Foley, N. M. et al. A genomic timescale for placental mammal evolution. *Science* 380, eabl8189; 10.1126/science.abl8189 (2023).

[16] Ronquist, F. et al. A total-evidence approach to dating with fossils, applied to the early radiation of the Hymenoptera. *Syst*. *Biol*. **61**, 973-999. (doi:10.1093/sysbio/sys058) (2012).

[17] Goloboff, P. A. Estimating character weights during tree search. *Cladistics* **9**, 83-91. (doi.org/10.1111/j.1096-0031.1993.tb00209.x) (1993).

[18] Chester, S. G. B., Williamson, T. E., Bloch, J. I., Silcox, M. T. & Sargis, E. J. Oldest skeleton of a plesiadapiform provides additional evidence for an exclusively arboreal radiation of stem primates in the Palaeocene. *R. Soc. Open Sci*. **4**, 170329; 10.1098/rsos.170329 (2017).

[19] Silcox, M. T., Bloch, J. I., Boyer, D. M. & Houde, P. Cranial anatomy of Paleocene and Eocene *Labidolemur kayi* (Mammalia: Apatotheria), and the relationships of the Apatemyidae to other mammals. *Zool*. *J*. *Linn*. *Soc*. **160**, 773-825. (doi:10.1111/j.1096-3642.2009.00614.x) (2010).

[20] Williamson, T. E. & Weil, A. Metatherian mammals from the Naashoibito Member, Kirtland Formation, San Juan Basin, New Mexico and their biochronologic and paleobiogeographic significance. *J. Vertebr. Paleontol.* **28**, 803-815. (doi: 10.1671/0272-4634(2008)28[803:MMFTNM]2.0.CO;2) (2008).

[21] Ogg, J. G. Geomagnetic polarity time scale in *The geologic time scale* 2012 (eds. Gradstein, F. M., Ogg, J. G., Schmitz, M. D. & Ogg, G.) 85-113 (Elsevier, 2012).

[22] Williamson, T. E. The beginning of the age of mammals in the San Juan Basin, New Mexico; biostratigraphy and evolution of Paleocene mammals of the Nacimiento Formation. *New Mexico Mus. Nat. Hist. Sci. Bull*. **8**, 1-141. (1996).
